# Variations in the structural and functional diversity of zooplankton over vertical and horizontal environmental gradients en route to the Arctic Ocean through the Fram Strait

**DOI:** 10.1371/journal.pone.0171715

**Published:** 2017-02-08

**Authors:** Marta Gluchowska, Emilia Trudnowska, Ilona Goszczko, Anna Maria Kubiszyn, Katarzyna Blachowiak-Samolyk, Waldemar Walczowski, Slawomir Kwasniewski

**Affiliations:** Institute of Oceanology Polish Academy of Sciences, Powstancow Warszawy Sopot, Poland; University of Shiga Prefecture, JAPAN

## Abstract

A multi-scale approach was used to evaluate which spatial gradient of environmental variability is the most important in structuring zooplankton diversity in the West Spitsbergen Current (WSC). The WSC is the main conveyor of warm and biologically rich Atlantic water to the Arctic Ocean through the Fram Strait. The data set included 85 stratified vertical zooplankton samples (obtained from depths up to 1000 metres) covering two latitudinal sections (76°30’N and 79°N) located across the multi-path WSC system. The results indicate that the most important environmental variables shaping the zooplankton structural and functional diversity and standing stock variability are those associated with depth, whereas variables acting in the horizontal dimension are of lesser importance. Multivariate analysis of the zooplankton assemblages, together with different univariate descriptors of zooplankton diversity, clearly illustrated the segregation of zooplankton taxa in the vertical plane. The epipelagic zone (upper 200 m) hosted plentiful, *Oithona similis*-dominated assemblages with a high proportion of filter-feeding zooplankton. Although total zooplankton abundance declined in the mesopelagic zone (200–1000 m), zooplankton assemblages in that zone were more diverse and more evenly distributed, with high contributions from both herbivorous and carnivorous taxa. The vertical distribution of integrated biomass (mg DW m^-2^) indicated that the total zooplankton biomass in the epipelagic and mesopelagic zones was comparable. Environmental gradients acting in the horizontal plane, such as the ones associated with different ice cover and timing of the spring bloom, were reflected in the latitudinal variability in protist community structure and probably caused differences in succession in the zooplankton community. High abundances of *Calanus finmarchicus* in the WSC core branch suggest the existence of mechanisms advantageous for higher productivity or/and responsible for physical concentration of zooplankton. Our results indicate that regional hydrography plays a primary role in shaping zooplankton variability in the WSC on the way to the Arctic Ocean, with additional effects caused by biological factors related to seasonality in pelagic ecosystem development, resulting in regional differences in food availability or biological production between the continental slope and the deep ocean regions.

## Introduction

Arctic amplification, caused by many factors operating on different time and space scales, is now recognised as an integral characteristic of the global climate system [1]. The factors causing arctic amplification are strongly linked but are not limited to the declining extent of sea ice and atmospheric and oceanic heat transports. Warm anomalies found all around the Arctic Ocean and a pronounced sea-ice minimum recorded in 2012 indicate that the Arctic is in transition towards a new warmer state [1–3]. The West Spitsbergen Current (WSC) provides the principal contribution of salt and sensible heat to the Arctic Ocean [4]. Therefore, knowledge of the physical and biological spatial heterogeneity of the WSC en route to the Arctic Ocean through the Fram Strait is a basic requirement for assessing the impact of climate change on the Arctic ecosystems, and can be used to create various scenarios dealing with global warming.

The Atlantic water (AW) is transported into the Nordic Seas by two branches of the Norwegian Atlantic Current [5–7]. The warmer, more saline eastern branch, known as the Norwegian Atlantic Slope Current, bifurcates after passing Norway into the stream of the AW, entering the Barents Sea and the core of the West Spitsbergen Current (WSCc) [8]. Due to bottom topography, the western branch of the Norwegian Atlantic Current flows along the Knipovich Ridge and converges with the WSCc as the WSC offshore branch (WSCo) in the region of the WSC between 77° and 77°30’N [5]. The complicated topographic structure of the Fram Strait leads to a splitting of the WSC in this region [9, 10]. A significant amount of the AW recirculates and returns south to the Nordic Seas as the Return Atlantic Water (RAW [11]), whereas only a part of the AW flow continues into the Arctic Ocean. The West Spitsbergen multi-path current system most likely highly influences the spatial biological heterogeneity of this region, especially in the pelagic realm.

Similar to other regions, plankton communities in the North Atlantic and Arctic Seas form assemblages with a close relationship to environmental variability, predominantly related to hydrography, resulting in diverse patterns in zooplankton distribution. Although the influx of AW to the Arctic Ocean has increased in recent decades [12–14], it remains unclear how this will affect the pelagic ecosystem. Zooplankton communities can be described with respect to their composition, structure, taxonomic indices and standing stock; these descriptors may be affected differently by the various drivers that exist, and this variability may depend on the spatial scale investigated, both in the horizontal and vertical dimensions. Over the past decade, there has been a growing recognition of the importance of relating community structure to ecosystem function in a wide range of ecological studies [15–19]. Although, the use of functional traits related to functional diversity (body size, ontogeny, habitat and feeding behaviours) potentially allows a more ecological point of view rather than a traditional taxonomic diversity [20], little attention has been paid to developing a functional diversity approach to marine zooplankton communities [21], especially in the Arctic. It is suggested that modification of the environmental conditions of the Arctic Ocean will be manifested in changes in the relative density and feeding strategies of some zooplankton species, in the age structures of populations and the biomass of communities [22] that occur due to changes in seasonality [23], temperature-dependent physiological constraints [24] and invasion by low-latitude allochthonous species [25–27].

At high latitudes, where primary production is highly seasonal [28], zooplankton communities are dominated by low number of species. In spring, these tend to be herbivorous, whereas in autumn omnivorous species tend to predominate [29]; nevertheless, a large overall variation in their diversity, evenness and distinctness is observed [30]. Recent study [25] has shown that the zooplankton in the WSC consist of a mixture of boreal, boreo-Arctic, Arctic and ubiquitous species, with apparent spatial and temporal variability in their occurrence. Although the zooplankton community in this region is numerically dominated by *Oithona similis*, the most important component in terms of biomass is *Calanus finmarchicus*. The available plankton data from the WSC region are highly fragmented in space and time, and the majority of these data are limited to the epipelagic zone [25, 29, 31–35]. Consequently, the spatial heterogeneity of zooplankton of the multi-path WSC system is still poorly understood.

The aim of this study was to determine which spatial environmental gradient, vertical or horizontal, is the most important in defining the distribution of zooplankton along the route to the Arctic Ocean through the Fram Strait. To that end, differences in zooplankton standing stock, community composition, the abundance of *C*. *finmarchicus* and its stage composition, as well as structural and functional zooplankton diversity, were compared along the vertical water column profiles between two latitudinal sections (76°30’N and 79°N) located across three longitudinal WSC regions. Of particular interest are the linkages between the hydrographic conditions, food availability and zooplankton community characteristics in the Atlantic-Arctic transition zone. In this study, we used for the first time the functional trait ‘trophic diversity’ in the subarctic Atlantic Ocean.

## Materials and methods

### Sampling

**Fig 1:**
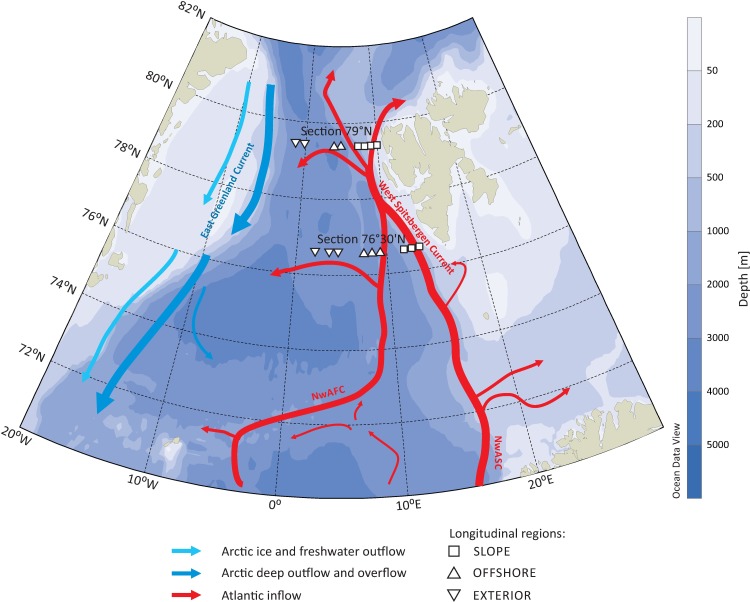
Overview map of the study area with circulation scheme after Beszczynska-Möller et al. [12] for the Nordic Seas and Fram Strait and location of sampling stations covering the two studied latitudinal sections and the three studied longitudinal regions of the WSC (SLOPE, OFFSHORE and EXTERIOR).

Sampling was conducted within a 2-week time window (13–21 July) during the IO PAN annual Arctic summer cruise in 2012 from aboard the RV ‘Oceania’. The sampling covered two horizontal gradients: latitudinal (LAT; two sections, southern at 76°30’N and northern at 79°N) and longitudinal (LON; three regions along the east-west line: easternmost on the west Spitsbergen slope area (SLOPE; 76°30’N– 3 stations; 79°N– 4 stations), westernmost area EXTERIOR (76°30’N– 3 stations; 79°N– 2 stations) and the central OFFSHORE area (76°30’N– 3 stations; 79°N– 2 stations). In total, 17 stations were established (Fig 1).

At each station, stratified vertical zooplankton hauls were made using a Multi Plankton Sampler type midi (Hydro-Bios, Germany) consisting of five closing nets with 0.25 m^2^ square openings and 0.180 mm mesh size gauze. Five fixed-depth strata (0-25-50-200-600-1000 m, or less if bottom was shallower) were sampled for evaluating zooplankton variability along vertical environmental gradient. The collected samples were preserved in 4% formaldehyde solution in seawater buffered with borax. A total of 85 samples was collected (S1 Table). The permission to conduct the study on that area was given by the Norwegian government. We confirm that the field studies did not involve endangered or protected species.

To analyse zooplankton food availability and quality, seawater samples were collected at each sampling station for the analysis of chlorophyll *a* concentration as well as protist biomass and composition. The samples were collected with Niskin bottles from 6 discrete depths (1, 5, 15, 25, 35, 50 m). For total chlorophyll *a* analysis, subsamples (250–500 ml) were filtered through Whatman GF/F glass-fibre filters and frozen immediately at -80°C. Seawater subsamples (200 ml) for protist identification and enumeration were fixed with alkaline Lugol’s solution and, after 24 h, with borax-buffered formaldehyde. Each fixative was added to 2% final concentration.

To characterise the physical properties of the water column, water temperature (°C) and salinity were derived from continuous measurements from the sea surface to the bottom at all zooplankton stations, and additionally at the hydrographic monitoring stations, along the LAT sections, using the Sea-Bird Electronics, Inc. CTD (SBE 911plus) system consisting of duplicated SBE 3plus premium temperature sensors, SBE 4C conductivity sensors, and a Digiquartz® pressure sensor.

### Sample processing

Zooplankton were identified to the lowest possible taxonomic level. Each sample was first scanned for macrozooplankton (organisms with total length >0.5 cm); these were picked out, identified and counted in the entire sample. The remaining mesozooplankton size fraction was examined for taxonomic composition and abundance by the subsampling method [36]. Subsamples of 2-ml volume were taken using a macropipette (an equivalent of the Stempel pipette), and all organisms in each subsample were identified and counted. The number of subsamples was determined individually to count at least 500 individuals per sample [36]. Representatives of *Calanus* were identified to the species level based on the description given in Kwasniewski et al. [37].

Chlorophyll *a* concentrations were measured fluorometrically [38] using a Perkin Elmer LS55 fluorescence spectrometer. Pigments from freeze-dried filters were extracted in 90% acetone for 24 h at 4°C. Emission at 671 nm after excitation at 431 nm was measured before and after sample acidification with 1 M HCl, and used to calculate the chlorophyll *a* concentration according to the method described in Utermöhl et al. [39].

Protist samples were qualitatively and quantitatively analysed using protocols described by Utermöhl [39] and modified by Edler [40]. A 10–50 ml subsample was placed in a counting chamber for 24 h; protists were then counted under an inverted microscope equipped with phase and interference contrast (Nikon Eclipse TE-300). For the most numerous taxa, 50 specimens were counted, and the number of fields of view was considered individually. With the exception of unidentified nanoplanktonic flagellates, each taxon was identified to the lowest possible taxonomic level and classified into a major taxonomic group in accordance with the World Register of Marine Species (WoRMS, http://www.marinespecies.org).

### Data analysis

Zooplankton abundance and biomass were expressed as the number of individuals or as mg of dry mass per cubic metre for each sampled depth-strata (ind m^-3^ and mg m^-3^, respectively). Zooplankton biomass was calculated from the abundance data and individual dry mass values or weight-length relationships obtained from the literature [33, 41–61]. When the horizontal pattern was investigated, zooplankton data were expressed as the average number of individuals or average biomass over a depth of 0–1000 m or of 0–50 m, or were integrated over the depth extension of the sampled layer, to assess the contribution of each layer to the total water column zooplankton biomass. Zooplankton taxa were also classified according to dietary preference (herbivorous, omnivorous and carnivorous) after Blachowiak-Samolyk et al. [29]; modified.

Various descriptors of zooplankton community structure, standing stock (total zooplankton abundance—TA and total zooplankton biomass—TB), and structural and functional diversity, as well as *C*. *finmarchicus* abundance, stage composition and stage index were used. Relative abundance of species/taxa in the sample was used for community structure determination. Hill’s diversity indices [62] were used to assess structural diversity because they reflect both species/taxa richness (H_o_) and evenness (H_inf_). The trophic diversity (TD) index [19, 63] modified for zooplankton communities was used as a functional diversity descriptor. The TD index was calculated based on the contribution of the biomass of each trophic group to the total zooplankton biomass; the reciprocal of TD was used (TD^-1^); thus, higher values correspond to higher trophic complexity. For the three trophic groups (herbivorous, omnivorous and carnivorous) identified in the present study, this index ranges from 1.0 to 3.0. The *C*. *finmarchicus* stage index was calculated as the abundance-weighted mean stage, with the stages assigned values from 1 (CI) to 6 (including abundance of both CVI females and males).

The protist biomass was estimated on the basis of the average carbon content of each cell given in the Nordic Microalgae web base (http://nordicmicroalgae.org). It was calculated using the volume-to-biomass formulas developed by Menden-Deuer and Lessard [64]. Chlorophyll *a* concentration and protist biomass were integrated for the upper 50 m of the water column and expressed as mg m^-2^ or mg C m^-2^, respectively.

CTD datasets were processed using SBE Data Processing Software. Profiles were averaged vertically with 1-m intervals. Further calculations and visualisation were performed in the MathWorks MATLAB environment. To support statistical analysis of the zooplankton data, water temperature and salinity were averaged for each sampled zooplankton depth-stratum.

Both univariate and multivariate non-parametric permutational ANOVAs (PERMANOVA; [65, 66]) were performed. Hydrographic parameters and the zooplankton data were analysed using 3-factor fixed model design with the following factors: latitude (LAT), longitude (LON) and water layer (WL). Differences in chlorophyll *a* concentration, total protist biomass and community structure were calculated using the 2-factor PERMANOVA test (LAT, LON). The calculation of pseudo-F and *p* values was based on 999 permutations of the residuals under a reduced model. To assess the magnitude of the spatial variation at each gradient, the estimated components of variation (ECV) as a percentage of the total variation were used.

The univariate descriptors included: water temperature and salinity, chlorophyll *a* concentration, total protist biomass, TA and TB, *C*. *finmarchicus* abundance and stage index, Hill’s indices and TD^-1^. All univariate tests were conducted on Euclidean distance similarity matrices. For multivariate analysis of the protist and zooplankton community structure as well as the *C*. *finmarchicus* stage composition, Bray-Curtis was used as a similarity measure to calculate the resemble matrix on relative abundance, square-root-transformed data.

A non-metrical multi-dimensional scaling plot (nMDS) was used to reveal and illustrate multivariate zooplankton community structure. To clearly visualise the spatial patterns of zooplankton variation, the nMDS ordination was plotted based on centroids (points located in the centre area of each group of points) of the three-way-interaction cell groupings (factors: LAT, LON, WL).

DistLM (distance-based linear model) routines were run to analyse and model the relationship between zooplankton species composition and environmental variables. Analyses were performed based on either the full data set (including data for all studied layers) or vertically averaged values over the sample depth 0–1000 m to investigate horizontal variability. A model for the upper 50-m layer was also constructed to analyse the relationships between herbivorous zooplankton (*C*. *finmarchicus*, *C*. *glacialis*, *C*. *hyperboreus*, *Pseudocalanus* spp., Copepoda nauplii, Cirripedia nauplii, *Apherusa glacialis*, *Thysanoessa inermis*, *T*. *longicaudata*, Euphausiacea nauplii, calyptopis and furcilia, *Limacina helicina*, Bryozoa larvae, *Fritillaria borealis*, *Oikopleura* cf. *vanhoeffeni*) and the hydrographic variables, chlorophyll *a* concentration and biomass of the dominant protist taxa. Relative zooplankton abundances were square-root-transformed prior to analysis. In all models, the forward selection procedure was used to determine the best combination of predictor variables, for explaining variation in zooplankton assemblages. The selection criteria chosen for the best-fitting relationship were based on *R*^*2*^ values [65]. Complementary to these analyses, non-parametric Spearman rank-order correlations were computed between selected univariate zooplankton characteristics (abundances of dominant species/taxa, TA and TB, *C*. *finmarchicus* stage index, structural and functional zooplankton diversity indices) and hydrographic variables.

All described statistical analyses were performed using PRIMER 6, PERMANOVA + [65, 67] and STATISTICA 10 (StatSoft, Inc.). The significance level for all statistical tests used was *p* = 0.05. The map of sampling stations was created with Ocean Data View 4 [68].

## Results

### Hydrographic environment

**Fig 2:**
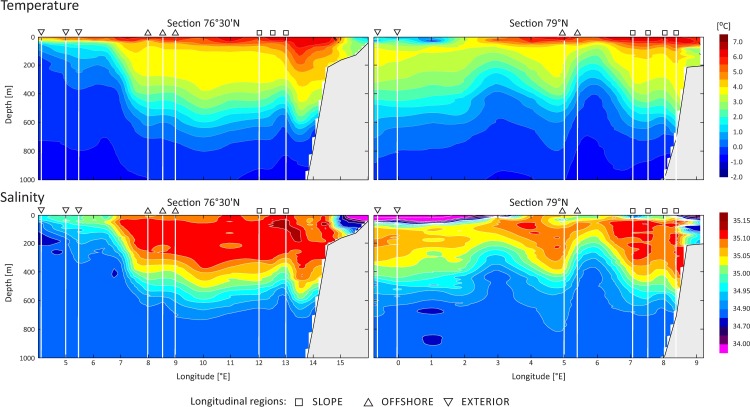
Temperature and salinity distributions in July 2012 in the southern 76°30’N and northern 79°N sections, with the locations of sampling stations covering the three studied longitudinal regions of the WSC (SLOPE, OFFSHORE and EXTERIOR).

Full-depth hydrographic measurements at the fixed stations revealed that the core of AW (WSCc) over the Spitsbergen slope reached 600 m at the LAT section 76°30’N (13°00’-13°30’E) and 500 m at the section 79°N (8°00’-8°40’E, Fig 2). Zooplankton stations in the SLOPE regions were collected from the western margin of the WSCc, but the water properties there were similar to the core, i.e., typical for the AW with a thin layer of very warm water at the top. In the LAT section 76°30’N, the WSCo (with weaker velocities [69]) appeared between 6°30’ and 7°30’E (Fig 2). Although zooplankton stations representing the OFFSHORE region were located east of 7°30’E, the water properties there were similar to the WSCo; therefore, these three stations can be regarded as representative of the WSCo. There was no RAW at that LAT section. The westernmost stations (3°40’-6°00’E, including region EXTERIOR) represented the Arctic Atlantic Water (AAW) flowing from the north and mixing on its way with AW and RAW. There was a thin (50-m) layer of the AW at the top of this flow. In the LAT section 79°N (Fig 2), zooplankton stations from the OFFSHORE region were located in a rotating structure. This was most likely created by anticyclonic meander of the slope current caused by bottom topography. At the westernmost stations (0°50’-3°00’E, region EXTERIOR), the RAW was covered by a thin layer of cold and fresh polar surface water. In the deeper layers, there was AAW.

The PERMANOVA results based on the average water temperature (T) and salinity (S) data (Table 1, S2 Table) showed significant differences among LAT sections (S), LON regions of the WSC (T) and WL (T, S). The average seawater temperature variability was the most pronounced among the water layers (WL; Table 1); it ranged from 1.41°C to 6.39°C in the upper 25 m (WL: 0–25 m) and from -0.51°C to 1.70°C below 600 m (WL: 600–1000 m). In both sections, water temperatures in the EXTERIOR were lower than in the OFFSHORE and SLOPE regions (Fig 2). In section 76°30’N, the differences were observed across the water column, whereas in section 79°N they were observed only in the upper 50-m layer.

**Table 1:** Two- and three-factor PERMANOVA results (%ECV—the estimated components of variation expressed as a percentage of the total variation) for the environmental and the zooplankton descriptor sets (univariate and multivariate).

		LAT	LON	WL	LATxLON	LATxWL	LONxWL	LATxLONxWL	Residual
**Hydrography**	*df*	1	2	4	2	4	8	8	54
Temperature	** **	1.1	18.1**	34.9**	2.9*	7.8**	11.4**	17.2**	6.5
Salinity	** **	7.9*	0.0	53.3**	4.8	0.0	0.0	7.6	26.4
**Pelagic protist**	*df*	1	2	-	2	-	-	-	11
Chlorophyll *a*	** **	0.0	13.2	-	0.0	-	-	-	86.8
Total biomass	** **	0.0	13.9	-	0.0	-	-	-	86.1
Community structure	** **	56.6*	4.7	-	0.0	-	-	-	38.7
**Zooplankton**	*df*	1	2	4	2	4	8	8	54
Standing stock–TA	** **	7.8*	0	53.3**	4.8	0.0	0.0	7.6	26.4
Standing stock–TB	** **	5.4	0.0	54.2**	4.2	0.0	0.0	8.3	27.8
Community structure	** **	12.5**	9.4**	31.1**	8.7**	10.8**	7.8**	0.0	19.4
*C*. *finmarchicus* abundance	** **	5.6*	10.4*	28.3*	2.3	0.0	13.2	0.0	40.0
*C*. *finmarchicus*—stage composition	** **	11.7*	9.9*	28.8*	3.4	13.3*	7.1	0.0	25.6
*C*. *finmarchicus*—stage index	** **	15.2*	11.6*	24.0*	0.0	19.7*	7.1	0.0	22.5
Structural diversity (richness)—H_o_	*** ***	0.0	0.0	51.3**	7.5*	3.6	9.2*	9.3	18.9
Structural diversity (evenness)—H_inf_	** **	18.5**	2.7	34.5**	0.0	17.9**	0.0	0.6	25.8
Trophic diversity—TD^-1^	*** ***	0.0	6.1	26.8**	12.1*	11.7	16.2**	0.0	26.9

LAT—latitudinal section; LON—longitudinal region; WL—water layer; df—degrees of freedom.

Significant effects: *p<0.05; **p<0.001.

Detailed results of PERMANOVA analysis are presented in S2 and S3 Tables.

Salinity showed lower variability than temperature (Table 1, S2 Table). In general, LAT section 79°N was characterised by slightly lower salinity (between 33.61 and 35.10) than section 76°30’N (between 34.90 and 35.15). The average salinity also varied significantly among water layers. High variation in salinity (33.61–35.13) was observed in surface waters, maximum values (34.90–35.15) were recorded in the 50–200 m layer, and the values decreased slightly and remained constant (34.91–35.07) below 200 m.

### Chlorophyll *a* and pelagic protist biomass

**Fig 3:**
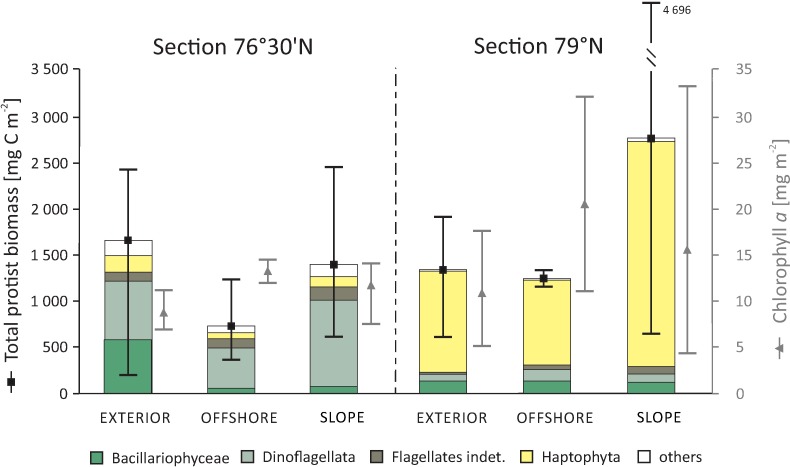
Pelagic protist biomass (mg C m-2) and concentration of chlorophyll a (mg m-2) integrated separately for the upper 50 m of the water column for each latitudinal section and longitudinal region. Mean and Min./Max. values are presented.

The PERMANOVA results indicated that differences in protist community structure were dependent on factors associated with LAT (Table 1, S2 Table). The major component of total biomass in section 79°N were Haptophyta (31.4–94.1%), represented mainly by *Phaeocystis pouchetii*, whereas at 76°30’N gross of the biomass was composed of Dinoflagellata (28.2–83.1%) and Bacillariophyceae (1.3–56.4%; Fig 3). Despite the differences in the protist community structure, there were no significant differences in the integrated (0–50 m) total pelagic protist biomass or chlorophyll *a* concentration, for any of the factors investigated (Table 1, S2 Table), most probably due to overall large variability. Across all the stations, the range of variation in total protist biomass was comparable, ranging from 204.9 to 4696.2 mg C m^-2^ (Fig 3). Chlorophyll *a* values were more variable in section 79°N (4.4–33.3 mg m^-2^) than in 76°30’N (7.4–14.5 mg m^-2^).

### Zooplankton

#### Standing stock

**Fig 4:**
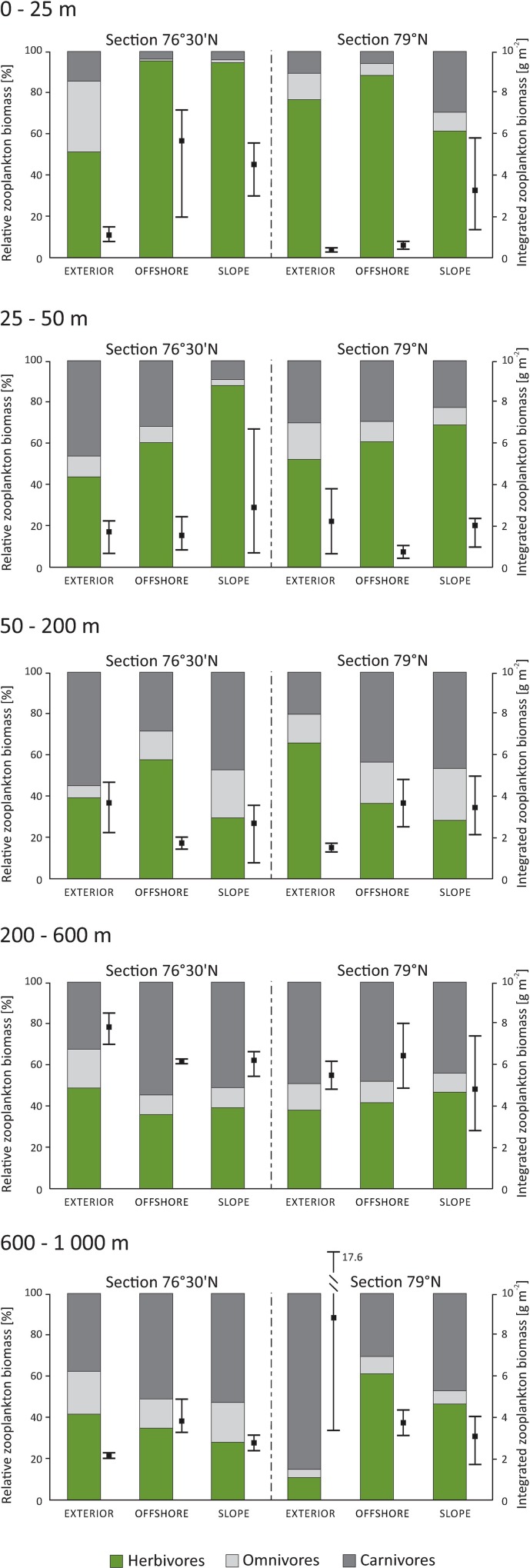
Vertical distribution of integrated zooplankton biomass (mg m^-2^) with contributions of different trophic levels indicated separately for each latitudinal section and longitudinal region. Mean and Min./Max. values of integrated zooplankton biomass are presented.

The highest variability in zooplankton standing stock values was associated with WL (Table 1). The highest TA and TB were recorded in the top 50-m layer, and the values usually decreased with depth (Table 2). Most of the zooplankton were concentrated above 50 m; this layer contained from 25 to 79% (mean 51%) of the total zooplankton abundance in the water column. The averaged vertical distribution of the water layer integrated biomass (mg DW m^-2^) indicated that a large portion of the zooplankton stock was concentrated in the upper 200 m (20.2–53.0% of the total biomass), but more than half of the total stock was present below 200 m (mean 59.4%, Fig 4) The total zooplankton biomass integrated over the entire sampled water column varied from 13.71 to26.00 g m^-2^.

**Table 2:** Mean and Min./Max. values of total zooplankton abundance (ind m^-3^), biomass (mg m^-3^), *C*. *finmarchicus* abundance (ind m^-3^) and stage index as well as values of selected structural (H_o_, H_inf_) and functional (TD^-1^) zooplankton community indices for established water layers (WL) and for latitudinal section (LAT) and longitudinal region (LON).

LAT	LON	WL	Zooplankton standing stock		*C*. *finmarchicus*		Structural diversity		Functional diversity
			Abundance -TA	Biomass—TB	Abundance	Stage index	Richness—H_o_	Evenness—H_inf_	Trophic diversity—TD^-1^
79°N	SLOPE	0–25	5423 (701–11218)	118 (55–230)	1446 (241–2724)	2.9 (2.1–4.4)	19 (15–23)	2.3 (1.7–2.9)	1.3 (1.2–1.4)
		25–50	2692 (1035–6400)	63 (38–94)	711 (132–1916)	3.1 (1.9–3.8)	21 (18–24)	2.4 (1.9–3.0)	1.9 (1.3–2.4)
		50–200	464 (370–579)	21 (14–33)	21 (6–38)	4.9 (4.4–5.6)	28 (26–29)	3.0 (2.6–3.6)	2.6 (2.3–2.8)
		200–600	100 (63–156)	12 (9–19)	20 (12–41)	4.9 (4.5–5.1)	34 (30–36)	4.1 (3.6–5.1)	2.3 (2.2–2.5)
		600–1000	48 (19–87)	12 (4–22)	15 (5–36)	4.9 (4.9–5.1)	34 (30–40)	3.1 (2.5–4.0)	2.2 (2.2–2.3)
	OFFSHORE	0–25	1646 (677–2617)	24 (16–32)	395 (119–672)	2.2 (2.0–2.4)	19 (17–20)	2.4 (2.1–2.7)	2.0 (1.8–2.3)
		25–50	1924 (1525–2323)	27 (17–38)	328 (188–470)	2.1 (2.1–2.2)	16 (15–16)	1.9 (1.4–2.4)	2.3 (1.9–2.7)
		50–200	518 (384–652)	25 (17–32)	24 (18–31)	4.5 (4.4–4.8)	27 (26–27)	2.9 (2.4–3.4)	2.8 (2.7–2.9)
		200–600	169 (153–185)	16 (12–20)	29 (24–34)	5.0 (5.0–5.1)	39 (37–40)	3.7 (3.7–3.8)	2.3 (2.3–2.3)
		600–1000	58 (24–92)	9 (8–11)	5 (5–6)	5.0 (5.0–5.0)	37 (30–44)	3.7 (3.0–4.4)	2.1 (1.8–2.4)
	EXTERIOR	0–25	1711 (1293–2130)	13 (11–14)	195 (86–304)	1.9 (1.5–2.4)	18 (16–20)	1.9 (1.7–2.1)	1.6 (1.6–1.6)
		25–50	2812 (2201–3423)	89 (26–153)	491 (447–537)	2.9 (2.0–3.9)	24 (21–26)	2.7 (2.3–3.1)	2.3 (1.9–2.6)
		50–200	487 (166–808)	10 (9–11)	110 (17–203)	3.2 (2.4–4.0)	23 (22–24)	3.2 (3.0–3.4)	2.1 (1.2–2.9)
		200–600	66 (158–175)	14 (12–15)	17 (17–19)	4.8 (4.7–5.1)	43 (42–43)	3.1 (2.9–3.3)	2.5 (2.4–2.5)
		600–1000	72 (68–76)	26 (8–44)	2 (2–3)	4.4 (4.0–4.9)	43 (41–45)	2.9 (2.6–3.3)	1.8 (1.2–2.4)
76°30’	SLOPE	0–25	1067 (610–1685)	169 (121–224)	541 (369–719)	5.2 (4.4–5.7)	21 (18–23)	2.0 (1.3–3.1)	1.1 (1.0–1.2)
		25–50	1825 (1389–2578)	111 (27–266)	420 (118–942)	3.9 (2.9–5.8)	22 (18–25)	1.4 (1.3–1.6)	1.8 (1.1–2.2)
		50–200	241 (143–327)	12 (5–34)	23 (5–59)	5.2 (4.9–5.9)	24 (22–27)	2.3 (1.7–3.1)	2.4 (1.8–2.9)
		200–600	74 (58–101)	16 (14–17)	15 (14–18)	5.0 (5.0–5.1)	38 (37–40)	3.7 (3.2–4.3)	2.3 (2.2–2.5)
		600–1000	26 (17–37)	7 (6–8)	2 (2–3)	4.8 (4.1–5.3)	35 (31–42)	3.2 (2.5–4.3)	2.5 (2.2–2.7)
	OFFSHORE	0–25	1499 (896–1871)	179 (79–289)	638 (182–876)	4.4 (3.8–5.1)	21 (18–22)	1.9 (1.5–2.1)	1.1 (1.1–1.3)
		25–50	1688 (1520–1775)	55 (33–92)	234 (130–302)	3.5 (2.8–3.9)	19 (17–21)	1.3 (1.2–1.4)	2.2 (1.9–2.4)
		50–200	321 (244–399)	11 (10–13)	12 (10–13)	4.8 (4.6–5.2)	26 (25–26)	1.7 (1.5–2.0)	2.7 (2.7–2.7)
		200–600	118 (96–148)	16 (15–16)	15 (15–17)	5.0 (5.0–5.1)	36 (33–39)	3.7 (2.7–4.6)	2.3 (2.2–2.4)
		600–1000	33 (19–52)	10 (8–12)	2 (2–4)	5.0 (5.0–5.1)	38 (33–42)	3.1 (2.4–4.3)	2.5 (2.2–2.8)
	EXTERIOR	0–25	6158 (397–13018)	41 (31–60)	175 (122–228)	3.4 (2.8–4.2)	20 (17–21)	1.8 (1.4–2.2)	2.1 (1.4–2.6)
		25–50	3754 (1494–5965)	65 (26–88)	158 (36–241)	4.0 (3.4–4.7)	20 (19–22)	1.4 (1.2–1.9)	2.4 (2.4–2.5)
		50–200	323 (257–371)	24 (15–32)	6 (5–10)	5.0 (4.9–5.1)	24 (23–26)	1.4 (1.2–1.5)	2.1 (2.1–2.2)
		200–600	77 (53–106)	19 (18–21)	1 (1–2)	4.4 (3.7–5.0)	36 (33–40)	3.6 (3.3–3.9)	2.6 (2.2–2.8)
		600–1000	19 (15–24)	5 (5–5)	1 (1–1)	3.4 (2.0–4.8)	34 (31–38)	3.1 (2.4–3.8)	2.7 (2.6–2.9)

Total zooplankton abundance also depended on factors associated with LAT sections (Table 1, S3 Table). The revealed pattern showed that the average total zooplankton abundance in the water column in section 79°N (311.4 ind m^-3^) was higher than in section 76°30’N (220.8 ind m^-3^). Across all the stations, the average total zooplankton biomass in the water layers was comparable (Table 1, S3 Table).

#### Zooplankton community structure

**Fig 5:**
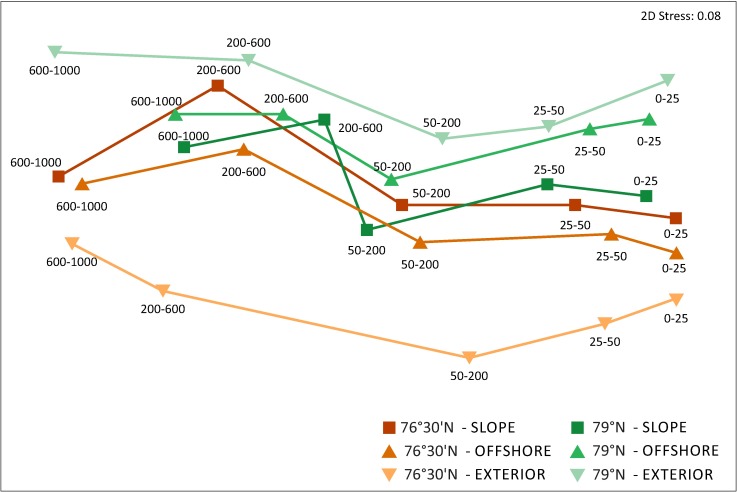
nMDS plot of centroids of Bray-Curtis similarity calculated for groups of samples collected from the same latitudinal section (LAT), longitudinal region (LON) and water layer. The numbers denote the thicknesses of the water layers in metres. The water layers for the same LAT and LON are connected by a line.

PERMANOVA analysis based on the zooplankton community indicated that differences in the zooplankton community structure were dependent on factors associated with LAT sections, LON regions and WL depth (Table 1, S3 Table). As in the case of standing stock, the community structure was first of all influenced by factors associated with WL (Table 1). A vertical gradient across the different WLs from the upper right of the plot (0–25 m) to the deep layers on the left (600–1000 m) was illustrated in the nMDS plot (Fig 5). Moreover, samples representing the southern section 76°30’N were placed in the lower part of the plot, whereas samples from the northern section 79°N were grouped within the upper part of the plot. The most visible separation of the sections were indicated in case of EXTERIOR regions.

**Fig 6:**
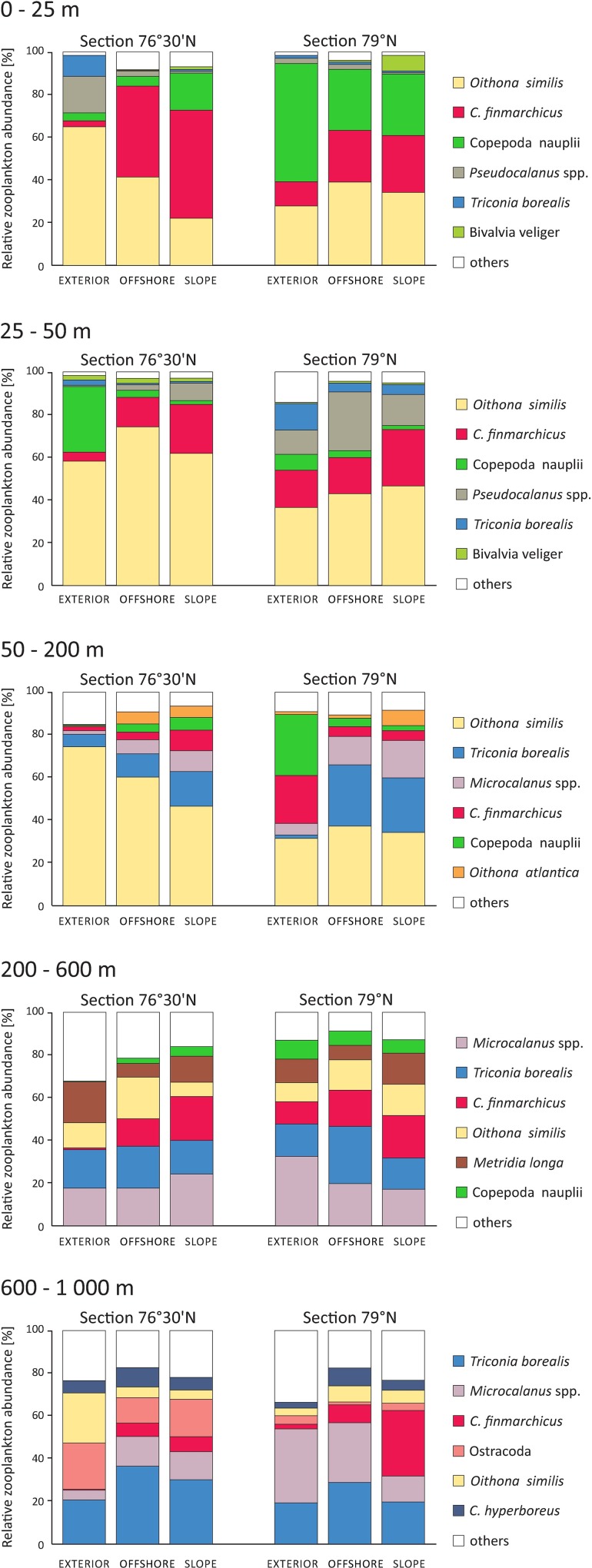
Vertical distribution of zooplankton community structure (six of the most important species/taxa were selected for each layer individually) for each latitudinal section and longitudinal region.

Altogether, the differences among WLs exceeded the differences between LAT sections and among LON regions (Table 1), indicating that the vertical heterogeneity is the main source of variation in the zooplankton structure in the region. Within 0–25 m and 25–50 m WLs, *O*. *similis*, *C*. *finmarchicus*, *Pseudocalanus* spp. and Copepoda nauplii were the dominant taxa of the zooplankton community in terms of abundance, with contributions ranging from 63 to 97% (mean 90%). In addition to the high proportion of *O*. *similis*, large shares of *Triconia borealis* and *Microcalanus* spp. were observed in the 50–200 m layer. Below 200 m, *T*. *borealis*, *Microcalanus* spp., *C*. *finmarchicus* and Ostracoda prevailed (Fig 6).

The differences among the LON regions were much greater for section 76°30’N than for section 79°N (Fig 6). In section 76°30’N, *C*. *finmarchicus* was present in the SLOPE and OFFSHORE regions at much higher relative abundance (4–51%) than in the EXTERIOR (0–4%), whereas the proportions of *O*. *similis* (WL 0–25 m, 600–1000 m), *Pseudocalanus* spp. (WL 0–25 m), *T*. *borealis* (WL 0–25 m) and Copepoda nauplii (WL 25–50 m) were higher in the EXTERIOR than in the SLOPE and OFFSHORE regions. In section 79°N, differences between regions were clearly observed only in WL 50–200 m, where *C*. *finmarchicus* and Copepoda nauplii constituted more than 50% of the total EXTERIOR community abundance and less than 10% in the SLOPE and OFFSHORE regions. An opposite trend was observed in the 600–1000 m layer; there, the highest relative abundances of *C*. *finmarchicus* were noted in the SLOPE region.

Generally, without taking different regions into account, differences between the LAT sections (Fig 6) were observed in the relative abundances of *O*. *similis* (WL 25–50 m, 50–200 m), *Pseudocalanus* spp. (WL 25–50 m) and Ostracoda (600–1000 m). *Pseudocalanus* spp. were present in higher proportions (11–28%) in section 79°N than in section 76°30’N (1–8%), whereas the contributions of *O*. *similis* and Ostracoda to the overall zooplankton abundance were lower in section 79°N than in section 76°30’N (31–47% to 47–74% *vs* 1–4% to 12–22%, respectively).

#### *C*. *finmarchicus* – abundance, stage composition, stage index

Based on ECV%, the most important causes of *C*. *finmarchicus* variability were environmental variables associated with WL depth strata, both in terms of abundance and stage composition (Table 1); however, there was also a significant variability caused by variables presumed to act on LAT sections influencing *C*. *finmarchicus* abundance and stage composition, and on LAT regions, influencing stage composition.

The highest abundance of *C*. *finmarchicus* was observed above 50 m (Table 2); when the horizontal dimension was examined, abundance was found to be higher in the northern section (79°N) than in the southern section (76°30’N) and higher in the SLOPE regions than in the EXTERIOR.

**Fig 7:**
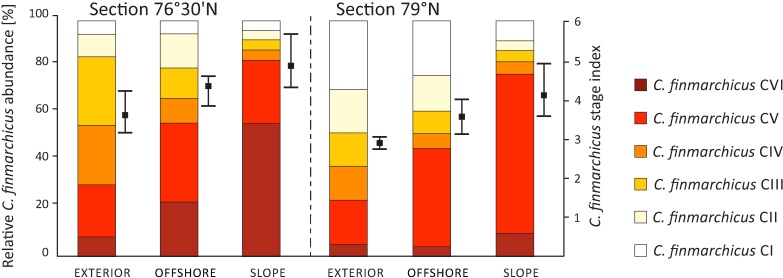
Stage composition and stage index of *C*. *finmarchicus* integrated separately for 0–1000 m depth for each latitudinal section and longitudinal region. Mean and Min./Max. values of the *C*. *finmarchicus* stage index are presented.

The *C*. *finmarchicus* stage index differed significantly among WL; however, due to the significant interactions LATxWL, the differences between layers were considered separately for the LAT sections. In LAT section 79°N, especially in the EXTERIOR and OFFSHORE regions, the population was composed predominantly of copepodids CI–CIII (stage index 1.5–3.9), which were concentrated in the upper 50 m, whereas more developed population (stage index 4.5–5.1) was found below 200 m. A more advanced developmental situation occurred in section 76°30’N (Table 2, Fig 7), with high contribution of CV and CVI in both the epipelagic and mesopelagic zones. There were also significant differences among LON regions, and in the SLOPE regions, *C*. *finmarchicus* CV and CVI accounted for 80% of the population (Fig 7).

#### Structural and trophic diversity of zooplankton

In our sample collection, a total of 78 taxa of 9 phyla were identified, including 63 species/genera and 15 taxa that were identified to higher taxonomic levels. Generally, Hill’s indices (H_o_, H_inf_) indicated that the upper 25 m (WL 0–25 m) harboured less diverse and less evenly distributed assemblages than the deeper layers (Table 2). Structural diversity showed a consistent increase in both indices’ values along the water depth, which was the most important contributor to the observed variability (Table 1). The PERMANOVA results indicated that across all stations, species diversity indices (H_o_) were similar (S3 Table) and ranged from 43 to 60 taxa, whereas the stations located in section 79°N were characterised by more even occurrence (H_inf_) of the noted taxa than the stations located in section 76°30’N.

Apart from an increasing trophic diversity towards the middle depth strata (Table 2), no obvious trends with respect to horizontal variability were observed. Herbivorous taxa dominated the assemblages in the upper 25 m, making up 79% of the total zooplankton biomass in that WL (Fig 4). The trophic structure showed a more even pattern in the deeper part of the water column, where the relative biomass of carnivores increased substantially, from 11% to 47% (Fig 4). The contribution of omnivorous taxa ranged from 10% to 18% of the community biomass over the entire water column.

### Relation between structure and function of zooplankton and environmental variables

The results of DistLM analyses showed that the hydrographic variables tested (temperature, salinity) explained 44% of the total variability in the abundance of zooplankton, when forward selection procedures were applied (S4 Table), with the water temperature having the highest impact on the observed variability (37%, ps-F = 47.74 *p* = 0.001). The abundance of dominant zooplankton taxa, as well as the TA and TB, scaled positively with temperature and/or salinity, whereas the *C*. *finmarchicus* stage index, as well as the structural and functional zooplankton community parameters (H_o_, H_inf_, TD^-1^), scaled negatively with hydrographic variables (Table 3).

**Table 3:** Spearman rank-order correlation coefficients (R) of zooplankton (abundances of dominant taxa, total zooplankton abundance (TA), total zooplankton biomass (TB), *C*. *finmarchicus* stage index as well as structural and functional community indices) in all layers and averaged over 0–1000 m, against hydrographic variables (T – temperature, S – salinity).

	all layers		avg. over 0–1000 m	
	T	S	T	S
*Calanus finmarchicus*	**0.817**	**0.400**	**0.603**	0.282
*Microcalanus* spp.	-0.182	0.131	0.297	0.142
*Pseudocalanu*s spp.	**0.679**	**0.295**	**-0.824**	-0.458
*Oithona similis*	**0.730**	**0.384**	-0.466	-0.211
*Triconia borealis*	**0.299**	0.159	-0.005	-0.248
Copepoda *nauplii*	**0.736**	**0.341**	0.331	-0.037
Zooplankton abundance (TA)	**0.778**	**0.371**	-0.086	-0.439
Zooplankton biomass (TB)	**0.706**	**0.342**	0.093	-0.250
*C*. *finmarchicus stage index*	**-0.289**	0.138	0.220	**0.674**
Species Richness—H_o_	**-0.717**	**-0.318**	-0.244	0.053
Species Evenness—H_inf_	**-0.440**	**-0.242**	0.336	0.027
Trophic diversity—TD^-1^	**-0.395**	-0.069	**-0.532**	-0.463

Bold values denote significance at *p*<0.05.

**Fig 8:**
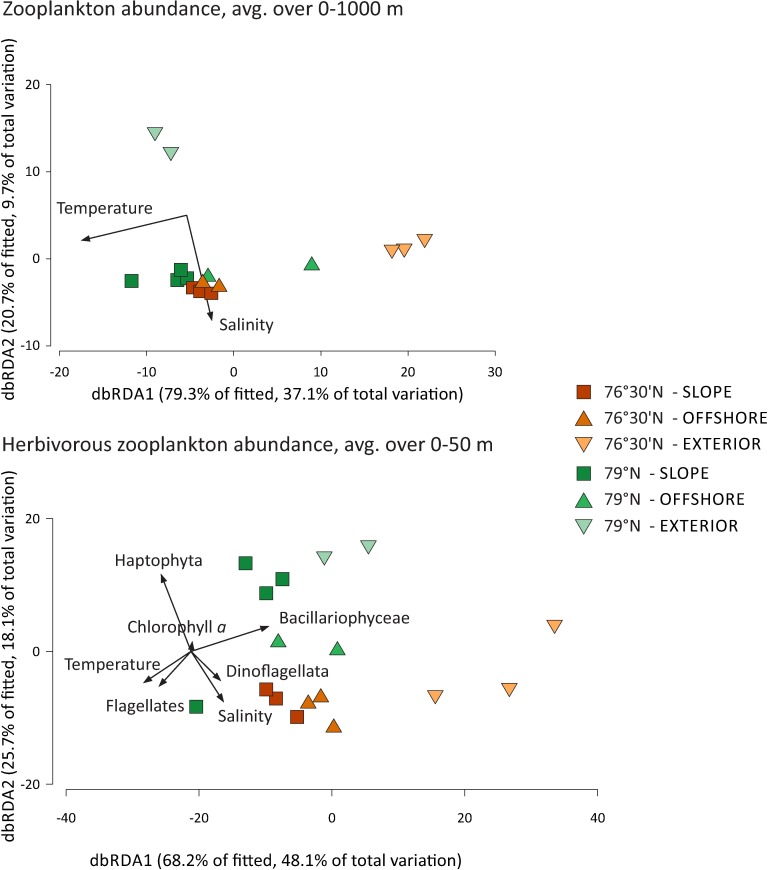
Distance-based redundancy (dbRDA) plots illustrating DistLM models of faunistic similarities among zooplankton and the relationship of zooplankton to hydrographic variables. The DistLM models presented are: layers integrated for 0–1000 m depth (upper) and layers integrated for 0–50 m depth (down). Model 0–1000 m depth was calculated for similarities among all zooplankton species/taxa; model 0–50 m depth was calculated for herbivorous species/taxa. Zooplankton abundance was square-root transformed; resemblance was estimated based on the Bray-Curtis similarity index. The axis legends include % of variation explained by the fitted model and % of total variation explained by the axis.

When only the horizontal dimension was examined (data for stations, averaged over 0–1000 m depth; Fig 8), the hydrographic variables explained 41% of the total zooplankton variability, of which a considerable portion (31%; ps-F = 6.50 *p* = 0.002) was explained by temperature (S4 Table). The abundance of *C*. *finmarchicus* was greater in warm water, and the population was older in the more saline waters (Table 3). TD^-1^ index values scaled negatively with temperature, indicating higher trophic complexity in colder and saltier waters.

In the case of the upper water layer (0–50 m), hydrographic variables, chlorophyll *a* concentration and the biomass of dominant protist taxa explained 59% of the total variation when the DistLM procedure was run (Fig 8) for herbivorous zooplankton communities (S4 Table). Among the tested variables, Bacillariophyceae biomass, temperature and salinity were statistically significant in the model. Herbivorous zooplankton variability was to a large extent controlled by Bacillariophyceae biomass (18% of the total variability; ps-F = 3.34 *p* = 0.012).

## Discussion

The aim of this study was to understand differences in zooplankton communities over vertical and horizontal gradients that represent different environmental variations en route to the Arctic Ocean through the Fram Strait. To achieve this goal, a set of stratified vertical zooplankton samples collected across the multi-path WSC system were analysed with respect to zooplankton community patterns, which were illustrated by the variability in zooplankton standing stocks and community structures, as well as by structural and functional diversity indices.

Among the investigated spatial gradients, the most important factors structuring zooplankton communities were those associated with depth. Because temperature and salinity also varied substantially in the vertical dimension (Table 1, Fig 2), the hydrographic gradient could be regarded responsible for the observed zooplankton variability. Nevertheless, the relationships between the zooplankton species assemblages and the relationships of the selected univariate zooplankton characteristics to the hydrographic variables indicated that only part of the variability reflects hydrographic conditions; thus, other factors that were not taken into consideration could also be involved. Vertical patterns of zooplankton distribution in the water column can be shaped by interconnected abiotic and biotic factors and processes, such as temperature, water masse type and transport, hydrographic stratification, pressure, light, seasonal and ontogenetic migration, food availability and predatory risk [70].

The highest total zooplankton abundances were generally found in the uppermost water layers and declined towards the deeper layers (Table 2). The overall pattern of zooplankton vertical distribution described in our survey is typical for the Arctic summer [71]. In most cases, zooplankton standing stocks are concentrated in the top 50 m [26, 29, 32, 71–74], and up to 80% of the zooplankton abundance may be restricted to the surface layer.

The vertical distribution patterns of the total zooplankton biomass were consistent with the patterns documented for zooplankton abundances and indicated that there was lower biomass per unit volume in the mesopelagic than in the epipelagic zone (Table 2). However, similarly to other studies [48, 71, 75], we observed also a shift towards larger (and consequently of higher biomass) zooplankton individuals in deeper water layers. This can be caused by changes in the zooplankton community composition and by the different morphometric characteristics of particular species and their patterns of occurrence (e.g., a decreasing number of smaller and thinner species such as *O*. *similis* at greater depths). Thus, the differences in zooplankton biomass between water layers were much lower than the differences in the abundances. In addition, when considering the vertical distribution of the biomass integrated for the individual water depths (mg m^-2^, Fig 4), we found that more than half of the zooplankton biomass was concentrated below 200 m. Transport of zooplankton to the Arctic Ocean is therefore equally important in the epipelagial (the upper 200-m layer) as in the mesopelagial (200–1000 m). Consequently, in the Arctic, where most studies are limited to the epipelagic zone [25, 35], the importance of unique assemblages of mesopelagic species is underestimated [71].

Multivariate analysis of the zooplankton assemblages and various univariate descriptors of its diversity clearly illustrated vertical segregation of zooplankton taxa (Table 1, Figs 5 and 6). An epipelagic zone of 200-m thickness hosted an *O*. *similis*-dominated aggregation with a high proportion of filter-feeding, predominantly herbivorous components (*C*. *finmarchicus*, *Pseudocalanus* spp. and Copepoda nauplii). The importance of small copepods, especially *O*. *similis*, in high latitude and Arctic ecosystems has been recognised also in previous studies [29, 35, 46, 74, 76]. These copepods, even though typically contributing little to zooplankton biomass, may play a pivotal role in secondary pelagic production [77, 78]. The most important in terms of biomass were herbivorous taxa (Fig 4), with *C*. *finmarchicus* as the prevailing species. At most stations, the abundance of this species peaked in the surface layers, which usually had high phytoplankton concentration. This suggests that a large fraction of *Calanus* was still actively feeding at the time of the study [79–81]. It is also worth mentioning that the populations of the species occupying surface waters, especially in the section 79°N, were dominated by early developmental stages. The mesopelagic zone was numerically dominated by *T*. *borealis*, *Microcalanus* spp. and older life stages of *C*. *finmarchicus* (Fig 6). High stock of *Oncaea* and *Triconia* (most likely *T*. *borealis*) has been previously observed in deep waters in the Arctic and sub-Arctic regions [59, 82, 83] year-round, probably due to close association of these cyclopoids with sinking marine aggregates [80, 83]. Because *Microcalanus* spp. are likely of Arctic origin [84], the greater observed abundance of these species in the coldest mesopelagic than in the epipelagic zone may be a result of seasonal migration to deeper water in early summer. The increased abundance of *C*. *finmarchicus* stage CV in the deeper water layers indicated that a portion of the population had probably begun its seasonal migration and initiated the descent to overwintering depths at the time of sampling [85, 86]. In terms of biomass, the most important species were *C*. *finmarchicus* and carnivorous taxa, mainly *Eukrohnia hamata*, *Paraeuchaeta* spp., *Aetideidae*, *Aglantha digitale*, Siphonophora and Ctenophora (data not shown).

The structural and trophic diversity indices increased with increasing water depth (Table 2) and were negatively correlated with temperature and salinity (Table 3). Previous studies reported that in the Canadian and Eurasian basins [71, 73], species diversity reached a maximum in the Atlantic water layer between 300 and 2000 m. In our study the upper layers were dominated by a few highly abundant omnivorous and herbivorous species, whereas in the mesopelagic zone we observed more diverse and more evenly distributed communities that showed high contributions from both herbivorous and carnivorous taxa. The highest biomass of herbivores near the surface and a predominance of carnivores in deeper water layers were observed previously in the Fram Strait [29], although that study was conducted during different seasons (spring and autumn) than our investigation. The share of omnivores in zooplankton biomass in summer 2012 was rather low in comparison with that in the two seasons analysed by Blachowiak-Samolyk et al. [29]. Our data fill an evident gap in available knowledge concerning the trophic structure of zooplankton en route to the Fram Strait.

Despite the fact that the vertical environmental gradient was of major importance in structuring zooplankton communities, large horizontal spatial gradients (latitudinal and longitudinal) that reflect broader oceanographic conditions also played a significant role in our survey. It is important to note that latitude and longitude *per se* are not the causes of ecological patterns. They have to be considered as proxies of currently unrevealed environmental factors, which act along the geographical gradients, and cause variability in zooplankton communities [87]. In the horizontal dimension, the key factors controlling pelagic diversity in the North Atlantic and adjacent seas seem to be water masses and sea currents [88–90]. Our study indicated that variability in water temperature and salinity had measurable effects on the zooplankton community in the study area, indicating that water mass distribution is important for the horizontal distribution of zooplankton in the study area. Temperature changed more with longitudinal regions, whereas salinity was more variable between latitudinal sections. Horizontal differences in water properties were the result of both the multi-path structure of the WSC in the western Spitsbergen region [5] and the natural transformation of the upper part of section 79°N into the less saline surface layer by melting sea ice and mixing with fresher surface water of Arctic origin [12]. Similarly to Reigstad et al. [91] and Nöthig et al. [92], we did not find large-scale differences in pelagic protist biomass or chlorophyll *a* concentration over the studied horizontal gradients; nevertheless, we observed differences in protist community structures (Fig 3).

Among the investigated horizontal gradients, latitude-related patterns were most important in structuring all characteristics of zooplankton structure, diversity and standing stock. One might argue that the latitudinal range sampled in this study is small (less than three degrees) and does not encompass wide variability. Nevertheless, previous hydrographic studies in the WSC region [5, 7] revealed that during its northward flow the AW undergoes intense transformation into less saline and colder waters, which most likely highly influenced the spatial biological heterogeneity. Differences in zooplankton communities between LAT sections were primarily caused by variations in species densities rather than by variations in taxonomic composition, which appears to be characteristic for the intermediate spatial scales (<1000 km [93]). High abundances of *C*. *finmarchicus* and *Pseudocalanus* spp. in section 79°N, together with relatively low abundance of *O*. *similis*, resulted in high species evenness in the northern stations. It is likely that latitudinal variability in protist community structure (Fig 3), probably resulting from differences in ice cover and/or timing of the spring bloom, causes differences in seasonal succession in the zooplankton community. Thus, *C*. *finmarchicus* development probably began earlier in the ice-free southern locations and therefore had progressed further than in the northern stations at the time of sampling (Fig 7). Our observations in the northern section at 79°N were consistent with the measurements of Nöthig et al. [92], which showed a massive bloom of the colonial form of the haptophyte (*P*. *pouchetii*) in the Fram Strait in 2012. *Pheocystis* blooms are not a new feature in this region; high abundances in the marginal ice zone were also reported during the 1980s and 1990s [94–96], in July and August of 2006 and 2007 in Kongsfjorden [97, 98], as well as in the Fram Strait [31, 98]. Recent studies on feeding strategies of *Calanus* [22] in the high Arctic suggest that the diet of *C*. *finmarchicus* consists not only of algae, as previously thought, but includes *Pheocystis* and small flagellates as equally important food sources. In addition, we discovered that communities in section 76°30’N (Fig 3) were dominated by microscopic heterotrophs (dinoflagellates). In recent years, several studies have noted the importance of flagellates in the Arctic [97, 99–102] and emphasised that climate change may influence the qualitative protist community structure by decreasing immobile diatom production and favouring motile nanoplanktonic phototrophs and microplanktonic heterotrophic protists.

Beaugrand et al. [103] reported pronounced local spatial variability in regional and global plankton diversity studies. Previous studies conducted in the WSC and adjacent seas [24, 25] that covered a larger longitudinal range (including the WSC, shelf areas of Spitsbergen, the Barents Sea and the Greenland Sea) also indicated substantial spatial differences in the zooplankton community composition and structure. However, in our study, the longitudinal environmental gradient in the WSC regions was rather weak and of local character. In previous studies, analysis of the spatial distribution of copepods, despite their importance in marine food webs, has mainly focused on the regional scale, and local variability could not be detected because of the weak resolution used. The observed spatial variability could also be related to the very complex and dynamic nature of the WSC in the studied region of the Fram Strait. The interaction with other ocean currents, as well as the recirculation process, results in substantial mixing of waters. Another important reason could be related to the generally heterogeneous nature of zooplankton distribution [104]. It is extremely difficult to assess zooplankton distribution on the local scale accurately using only traditional sampling methods [105]. High-resolution *in situ* measurements using a laser optical plankton counter have provided insight into the substantial role of zooplankton patchiness in the dynamic WSC region; this patchiness was either related to environmental discontinuities (eddies, frontal systems, water column stratification) or to assumed ecological niche portioning [69]. The observed temperature changes along the studied sections reflected differences between water masses present in the studied area, ranging from low values in the EXTERIOR regions to high temperatures in the SLOPE. Based on planktonic protist biomass and community structure, we could not separate longitudinal regions along east-west sections. Our results support the conclusions of Cherkasheva et al. [106], which indicate the relative homogeneity of phytoplankton distribution in the Fram Strait open ocean zone. However, the occurrence of horizontal gradients of temperature in the multi-path WSC system, together with barotropic and baroclinic instabilities that probably develop due to the presence of topographic discontinuities [107, 108], seems to have an impact on zooplankton variability and affect their structure and development. The SLOPE region, located at the eastern margin of the WSCc, was characterised mainly by a high relative abundance of *C*. *finmarchicus*. This suggests that the advective transport is the most important mechanism regulating the abundance of this species in the study area at the edge of the distribution [109] and underlines the notion that the flow of the WSC is an important conveyer of *C*. *finmarchicus* into the Arctic. The higher *C*. *finmarchicus* abundance along the slope may also result from the influence of the West Spitsbergen Shelf frontal system and from interactions between Arctic- and Atlantic-origin water masses that, by particle concentration and retention, provide conditions for enhanced biological productivity during summer [90, 110–114]. The other end of the studied environmental gradient spans the EXTERIOR region, representing the mix of Arctic Atlantic Water flowing from the north with AW, which is characterised by lower water temperature and salinity [115, 116]. The zooplankton community in this region was characterised by higher abundances of the Arctic-associated copepods *C*. *hyperboreus* and *Pseudocalanus* spp. (most probably *P*. *minutus*). The abundance of *Pseudocalanus* spp. scaled negatively with water temperature (Table 3), confirming that *P*. *minutus* is more numerous where mixing between Atlantic and Arctic waters takes place [117]. Differences between regions were significant not only in terms of *C*. *finmarchicus* abundance but also with respect to the stage composition and consequently the stage index of this species. It is therefore likely that variability in water temperature between the regions has resulted in seasonal succession in the zooplankton community. In the SLOPE region, where the temperature of the upper 50-m layer was significantly higher than in the EXTERIOR (approx. 2°C in section 76°30’N and 4°C in section 79°N, Table 3), *C*. *finmarchicus* CV and CVI represented 80% of the population (Fig 7), whereas in the EXTERIOR the population was composed of predominantly copepodids CI–CIII. According to [32], the development of *C*. *finmarchicus* may not be limited only by temperature *per se*; the timing of food availability may also be important.

The results of the present study indicate that en route to the Arctic Ocean through the Fram Strait, variations in the structural and functional diversity of zooplankton are greater over vertical than horizontal environmental gradients. Both univariate and multivariate analysis of zooplankton assemblages indicated that if we are to improve our understanding of zooplankton patterns in the horizontal plane, we need to focus on different water layers separately. The present study well documents that the main role in shaping the observed zooplankton variability in the WSC system is played by regional hydrodynamic processes, with additional effects related to biological cues such as seasonality and food availability. To fully understand and predict the effects of climate fluctuations on the WSC system, future studies should investigate how the environmentally driven variability in zooplankton community structure affects zooplankton function. Taxonomic and functional diversity indices represent complementary and reliable approaches, and functional diversity indices that take into account not only trophic group but also feeding type, body length, habitat type, vertical distribution preference, etc. may be more useful tools than the taxonomic approach for capturing subtle changes in community structure and function in a changing ocean.

## Supporting information

S1 Table Information on sample collection, July 2012. LAT – latitudinal sections; LON – longitudinal regions; WL – water layer extent.
(DOCX)

S2 Table Two- and three-factor PERMANOVA results for the environmental descriptor sets and 999 permutations. LAT – latitudinal section; LON – longitudinal region; WL – water layer; df – degrees of freedom; MS – means of squares; √ECV – square root of estimated components of variance; %ECV – percentage of ECV to total variation. Bold values denote significance at *p*<0.05.
(DOCX)

S3 Table Three-factor PERMANOVA results for the zooplankton descriptor sets and 999 permutations. LAT – latitudinal section; LON – longitudinal region; WL – water layer; df – degrees of freedom; MS – means of squares; √ECV – square root of estimated components of variance; %ECV – percentage of ECV to total variation. Bold values indicate *p*<0.05.
(DOCX)

S4 Table Results of the DistLM procedure for fitting hydrographic variables, chlorophyll *a* concentration and biomass of dominant protist taxa to the zooplankton community (model 0–50 m only herbivorous species). Var%—percentage of explained variance; Cum%—cumulative percentage explained by the added variable. Bold values denote significance at *p*<0.05.
(DOCX)

## References

[1] Serreze MC, Barry RG. Processes and impacts of Arctic amplification: A research synthesis. Global and Planetary Change. 2011;77:85–96.

[2] Polyakov IV, Beszczynska A, Carmack EC, Dmitrenko IA, Fahrbach E, Frolov IE, et al One more step toward a warmer Arctic. Geophysical Research Letters. 2005;32:L17605.

[3] Miles MW, Divine DV, Furevik T, Jansen E, Moros M, Ogilvie AE. A signal of persistent Atlantic multidecadal variability in Arctic sea ice. Geophysical Research Letters. 2014;41:463–9.

[4] Aagaard K, Foldvik A, Hillman S. The West Spitsbergen Current: disposition and water mass transformation. Journal of Geophysical Research. 1987;92(C4):3778–84.

[5] Walczowski W, Piechura J, Osinski R, Wieczorek P. The West Spitsbergen Current volume and heat transport from synoptic observations in summer. Deep Sea Research I. 2005;52:1374–91.

[6] Walczowski W, Piechura J. Pathways of the Greenland Sea warming. Geophysical Research Letters. 2007;34(L10608):1–5.

[7] Walczowski W, Piechura J. Influence of the West Spitsbergen Current on the local climate. International Journal of Climatology. 2011;31:1088–93.

[8] Nøst OA, Isachsen PE. The large-scale time-mean ocean circulation in the Nordic Seas and Arctic Ocean estimated from simplified dynamics. Journal of Marine Research. 2003;61:175–210.

[9] Perkin R, Lewis E. Mixing in the West Spitsbergen current. Journal of Physical Oceanography. 1984;14(8):1315–25.

[10] Quadfasel D, Gascard JC, Koltermann KP. Large‐scale oceanography in Fram Strait during the 1984 Marginal Ice Zone Experiment. Journal of Geophysical Research. 1987;92:6719–28.

[11] Bourke R, Weigel A, Paquette R. The westward turning branch of the West Spitsbergen Current. Journal of Geophysical Research. 1988;93:14065–77.

[12] Beszczynska-Möller A, Fahrbach E, Schauer U, Hansen E. Variability in Atlantic water temperature and transport at the entrance to the Arctic Ocean, 1997–2010. ICES Journal of Marine Science. 2012;69(5):852–63.

[13] Morison J, Aagaard K, Steele M. Recent environmental changes in the Arctic: a review. Arctic. 2000:359–71.

[14] Schauer U, Fahrbach E, Osterhus S, Rohardt G. Arctic warming through the Fram Strait: Oceanic heat transport from 3 years of measurements. Journal of Geophysical Research. 2004;109(C06026):1–14.

[15] Biswas SR, Mallik AU. Disturbance effects on species diversity and functional diversity in riparian and upland plant communities. Ecology. 2010;91:28–35. 20380192 10.1890/08-0887.1

[16] Schittko C, Hawa M, Wurst S. Using a multi-trait approach to manipulate plant functional diversity in a biodiversity-ecosystem function experiment. PLoS ONE 9(6): e99065 10.1371/journal.pone.0099065 24897501 PMC4045913

[17] Stuart-Smith RD, Bates AE, Lefcheck JS, Duffy JE, Baker SC, Thomson RJ, et al Integrating abundance and functional traits reveals new global hotspots of fish diversity. Nature. 2013;501:539–42. 10.1038/nature12529 24067714

[18] Paganelli D, Marchini A, Occhipinti-Ambrogi A. Functional structure of marine benthic assemblages using Biological Traits Analysis (BTA): a study along the Emilia-Romagna coastline (Italy, North-West Adriatic Sea). Estuarine, Coastal and Shelf Science. 2012;96:245–56.

[19] Ingels J, Vanreusel A. The importance of different spatial scales in determining structural and functional characteristics of deep-sea infauna communities. Biogeosciences. 2013;10(7):4547–63.

[20] Litchman E, Ohman MD, Kiørboe T. Trait-based approaches to zooplankton communities. Journal of Plankton Research. 2013:1–12.

[21] Pomerleau C, Sastri AR, Beisner BE. Evaluation of functional trait diversity for marine zooplankton communities in the Northeast subarctic Pacific Ocean. Journal of Plankton Research. 2015;37(4):712–26.

[22] Søreide JE, Falk-Petersen S, Hegseth EN, Hop H, Carroll ML, Hobson KA, et al Seasonal feeding strategies of Calanus in the high-Arctic Svalbard region. Deep Sea Research Part II. 2008;55:2225–44.

[23] Edwards M, Richardson AJ. Impact of climate change on marine pelagic phenology and trophic mismatch. Nature. 2004;430:881–4. 10.1038/nature02808 15318219

[24] Carstensen J, Weydmann A, Olszewska A, Kwaśniewski S. Effects of environmental conditions on the biomass of Calanus spp. in the Nordic Seas. Journal of Plankton Research. 2012;34:951–66.

[25] Weydmann A, Carstensen J, Goszczko I, Dmoch K, Olszewska A, Kwasniewski S. Shift towards the dominance of boreal species in the Arctic: inter-annual and spatial zooplankton variability in the West Spitsbergen Current. Marine Ecology Progress Series. 2014;501:41–52.

[26] Kosobokova K, Hirche H-J. Zooplankton distribution across the Lomonosov Ridge, Arctic Ocean: species inventory, biomass and vertical structure. Deep Sea Research Part I. 2000;47:2029–60.

[27] Carmack E, Wassmann P. Food webs and physical–biological coupling on pan-Arctic shelves: unifying concepts and comprehensive perspectives. Progress in Oceanography. 2006;71:446–77.

[28] Falk-Petersen S, Hop H, Budgell WP, Hegseth EN, Korsnes R, Løyning TB, et al Physical and ecological processes in the marginal ice zone of the northern Barents Sea during the summer melt period. Journal of Marine Systems. 2000;27:131–59.

[29] Blachowiak-Samolyk K, Kwasniewski S, Dmoch K, Hop H, Falk-Petersen S. Trophic structure of zooplankton in the Fram Strait in spring and autumn 2003. Deep Sea Research Part II. 2007;54:2716–28.

[30] Woodd-Walker RS, Ward P, Clarke A. Large-scale patterns in diversity and community structure of surface water copepods from the Atlantic Ocean. Marine Ecology Progress Series. 2002;236:189–203.

[31] Hop H, Falk-Petersen S, Svendsen H, Kwasniewski S, Pavlov V, Pavlova O, et al Physical and biological characteristics of the pelagic system across Fram Strait to Kongsfjorden. Progress in Oceanography. 2006;71(2):182–231.

[32] Hirche H-J, Kosobokova K. Distribution of Calanus finmarchicus in the northern North Atlantic and Arctic Ocean—expatriation and potential colonization. Deep Sea Research II. 2007;54:2729–47.

[33] Hirche H-J, Baumann M, Kattner G, Gradinger R. Plankton distribution and the impact of copepod grazing on primary production in Fram Strait, Greenland Sea. Journal of Marine Systems. 1991;2(3):477–94.

[34] Blachowiak-Samolyk K, Søreide JE, Kwasniewski S, Sundfjord A, Hop H, Falk-Petersen S, et al Hydrodynamic control of mesozooplankton abundance and biomass in northern Svalbard waters (79–81 N). Deep Sea Research Part II. 2008;55:2210–24.

[35] Svensen C, Seuthe L, Vasilyeva Y, Pasternak A, Hansen E. Zooplankton distribution across Fram Strait in autumn: Are small copepods and protozooplankton important? Progress in Oceanography. 2011;91:534–44.

[36] Harris R, Wiebe P, Lenz J, Skjoldal H-R, Huntley M. ICES zooplankton methodology manual: Academic Press; 2000.

[37] Kwasniewski S, Hop H, Falk-Petersen S, Pedersen G. Distribution of *Calanus* species in Kongsfjorden, a glacial fjord in Svalbard. Journal of Plankton Research. 2003;25:1–20.

[38] Evans CA, O'Reilly JE. A Manual for the Measurement of Chlorophyll A, Net Phytoplankton, and Nannoplankton: BIOMASS; 1983. 44p.

[39] Utermöhl H. Zur Vervollkommnung der quantitativen phytoplankton-methodik: E. Schweizerbart'sche; 1958.

[40] Edler L. Recommendations on methods for marine biological studies in the Baltic Sea Phytoplankton and Chlorophyll: The Baltic Marine Biologists Publications, University of Lund.

[41] Berestovskii E, Anisimova N, Denisenko C, Luppova E, Savinov V, Timofeev C. Relationships between size and body mass of some invertebrates and fish of the North-East Atlantic. Academy of Sciences of the USSR, Murman Marine Biological Institute, Apatity 1989.

[42] Deibel D. Feeding mechanism and house of the appendicularian *Oikopleura vanhoeffeni*. Marine Biology. 1986;93(3):429–36.

[43] Fenaux R. Cycle vital, croissance et production chez *Fritillaria pellucida* (Appendicularia), dans la baie de Villefranche-sur-Mer, France. Marine Biology. 1976;34(3):229–38.

[44] Hansen BW, Nielsen TG, Levinsen H. Plankton community structure and carbon cycling on the western coast of Greenland during the stratified summer situation: III. Mesozooplankton. Aquatic Microbial Ecology. 1999;16(3):233–49.

[45] Hay S, Kiørboe T, Matthews A. Zooplankton biomass and production in the North Sea during the autumn circulation experiment, October 1987–March 1988. Continental Shelf Research. 1991;11(12):1453–76.

[46] Hopcroft R, Clarke C, Nelson R, Raskoff K. Zooplankton communities of the Arctic’s Canada Basin: the contribution by smaller taxa. Polar Biology. 2005;28(3):198–206.

[47] Hopcroft R, Roff J, Webber M, Witt J. Zooplankton growth rates: the influence of size and resources in tropical marine copepodites. Marine Biology. 1998;132(1):67–77.

[48] Hopcroft RR, Kosobokova KN, Pinchuk AI. Zooplankton community patterns in the Chukchi Sea during summer 2004. Deep Sea Research Part II. 2010;57(1):27–39.

[49] Hygum B, Rey C, Hansen B. Growth and development rates of *Calanus finmarchicus* nauplii during a diatom spring bloom. Marine Biology. 2000;136(6):1075–85.

[50] Hygum B, Rey C, Hansen BW, Carlotti F. Rearing cohorts of *Calanus finmarchicus* (Gunnerus) in mesocosms. ICES Journal of Marine Science. 2000;57(6):1740–51.

[51] Auel H, Werner I. Feeding, respiration and life history of the hyperiid amphipod Themisto libellula in the Arctic marginal ice zone of the Greenland Sea. Journal of Experimental Marine Biology and Ecology. 2003;296(2):183–97.

[52] Båmstedt U. Chemical composition and energy content The biological chemistry of marine copepods Clarendon Press, Oxford 1986:1–58.

[53] Båmstedt U, Eilertsen HC, Tande KS, Slagstad D, Skjoldal HR. Copepod grazing and its potential impact on the phytoplankton development in the Barents Sea. Polar Research. 1991;10(2):339–54.

[54] Hansen BW, Bjørnsen P, Hansen B. Zooplankton grazing and growth: Scaling within the 2–2,000-μm body size range. Limnology and Oceanography. 1997;42:687–704.

[55] Ikeda T, Shiga N. Production, metabolism and production/biomass(P/B) ratio of *Themisto japonica* (Crustacea: Amphipoda) in Toyama Bay, southern Japan Sea. Journal of Plankton Research. 1999;21(2):299–308.

[56] Yamaguchi A, Ikeda T. Vertical distribution, life cycle, and developmental characteristics of the mesopelagic calanoid copepod *Gaidius variabilis* (Aetideidae) in the Oyashio region, western North Pacific Ocean. Marine Biology. 2000;137(1):99–109.

[57] Mumm N. On the summerly distribution of mesozooplankton in the Nansen Basin, Arctic Ocean. Berichte zur Polarforschung. 1991;92.

[58] Poltermann M. Biology and ecology of cryopelagic amphipods from Arctic sea ice. Berichte zur Polarforschung. 1997;225.

[59] Richter C. Regional and seasonal variability in the vertical distribution of mesozooplankton in the Greenland Sea. Berichte zur Polarforschung. 1994;154:1–87.

[60] Turner JT, Levinsen H, Nielsen TG, Hansen BW. Zooplankton feeding ecology: grazing on phytoplankton and predation on protozoans by copepod and barnacle nauplii in Disko Bay, West Greenland. Marine Ecology Progress Series. 2001;221:209–19.

[61] Matthews J, Hestad L. Ecological studies on the deep-water pelagic community of Korsfjorden, Western Norway: length/weight relationships for some macroplanktonic organisms. Sarsia. 1977;63(1):57–63.

[62] Hill MO. Diversity and evenness: a unifying notation and its consequences. Ecology. 1973;54(2):427–32.

[63] Heip C, Vincx M, Vranken G. The ecology of marine nematodes: Oceanigr. Marine Biology; 1985. 399–489 p.

[64] Menden-Deuer S, Lessard EJ. Carbon to volume relationships for dinoflagellates, diatoms, and other protist plankton. Limnology and Oceanography. 2000;45(3):569–79.

[65] Anderson M, Gorley RN, Clarke RK. Permanova+ for Primer: Guide to Software and Statistical Methods. 2008.

[66] Anderson M, Gorley R, Clarke R. Permanova. Permutational multivariate analysis of variance, a computer program Department of Statistics, University of Auckland 2005.

[67] Clarke K, Warwick RM. PRIMER v5: User manual: PRIMER-E Limited; 2001.

[68] Schlitzer R. Ocean Data View, http://odv.awi.de 2016.

[69] Trudnowska E, Gluchowska M, Beszczynska-Moller A, Blachowiak-Samolyk K, Kwasniewski S. Plankton patchiness in the upper layer of the Polar Front region, west of Spitsbergen. Marine Ecology Progress Series. 2016;560:1–18.

[70] Banse K. On the vertical distribution of zooplankton in the sea. Progress in Oceanography. 1964;2:53–125.

[71] Kosobokova KN, Hopcroft RR. Diversity and vertical distribution of mesozooplankton in the Arctic's Canada Basin. Deep Sea Research Part II. 2010;57:96–110.

[72] Sameoto D. Vertical distribution of zooplankton biomass and species in northeastern Baffin Bay related to temperature and salinity. Polar Biology. 1984;2(4):213–24.

[73] Kosobokova K, Hirche H-J. Biomass of zooplankton in the eastern Arctic Ocean–a base line study. Progress in Oceanography. 2009;82:265–80.

[74] Auel H, Hagen W. Mesozooplankton community structure, abundance and biomass in the central Arctic Ocean. Marine Biology. 2002;140:1013–21.

[75] Blachowiak-Samolyk K, Kwasniewski S, Richardson K, Dmoch K, Hansen E, Hop H, et al Arctic zooplankton do not perform diel vertical migration (DVM) during periods of midnight sun. Marine Ecology Progress Series. 2006;308:101–16.

[76] Lane PV, Llinás L, Smith SL, Pilz D. Zooplankton distribution in the western Arctic during summer 2002: hydrographic habitats and implications for food chain dynamics. Journal of Marine Systems. 2008;70(1):97–133.

[77] Turner JT. The importance of small planktonic copepods and their roles in pelagic marine food webs. Zool Stud. 2004;43(2):255–66.

[78] Gallienne C, Robins D. Is *Oithona* the most important copepod in the world's oceans? Journal of Plankton Research. 2001;23(12):1421–32.

[79] Eilertsen H, Tande K, Taasen J. Vertical distributions of primary production and grazing by *Calanus glacialis* Jaschnov and *C*. *hyperboreus* Krøyer in Arctic waters (Barents Sea). Polar Biology. 1989;9(4):253–60.

[80] Thor P, Nielsen T, Tiselius P, Juul-Pedersen T, Michel C, Møller E, et al Post-spring bloom community structure of pelagic copepods in the Disko Bay, Western Greenland. Journal of Plankton Research. 2005;27(4):341–56.

[81] Daase M, Eiane K, Aksnes DL, Vogedes D. Vertical distribution of *Calanus* spp. and *Metridia longa* at four Arctic locations. Marine Biology Research. 2008;4(3):193–207.

[82] Estrada R, Harvey M, Gosselin M, Starr M, Galbraith PS, Straneo F. Late-summer zooplankton community structure, abundance, and distribution in the Hudson Bay system (Canada) and their relationships with environmental conditions, 2003–2006. Progress in Oceanography. 2012;101:121–45.

[83] Gislason A. Life-cycle strategies and seasonal migrations of oceanic copepods in the Irminger Sea2003.

[84] Head E, Harris L, Yashayaev I. Distributions of *Calanus* spp. and other mesozooplankton in the Labrador Sea in relation to hydrography in spring and summer (1995–2000). Progress in Oceanography. 2003;59:1–30.

[85] Pedersen G, Tande K, Ottesen G. Why does a component of *Calanus finmarchicus* stay in the surface waters during the overwintering period in high latitudes? ICES Journal of Marine Science. 1995;52(3–4):523–31.

[86] Hirche H-J. Diapause in the marine copepod, *Calanus finmarchicus*—a review. Ophelia. 1996;44:129–43.

[87] Hawkins BA, Diniz‐Filho F, Alexandre J. ‘Latitude’and geographic patterns in species richness. Ecography. 2004;27(2):268–72.

[88] Beaugrand G, Ibañez F, Lindley JA, Reid PC. Diversity of calanoid copepods in the North Atlantic and adjacent seas: species associations and biogeography. Marine Ecology Progress Series. 2002;232:179–95.

[89] Yebra L, Harris R, Head E, Yashayaev I, Harris L, Hirst A. Mesoscale physical variability affects zooplankton production in the Labrador Sea. Deep Sea Research Part I. 2009;56(5):703–15.

[90] Garçon VC, Oschlies A, Doney SC, McGillicuddy D, Waniek J. The role of mesoscale variability on plankton dynamics in the North Atlantic. Deep Sea Research Part II. 2001;48(10):2199–226.

[91] Reigstad M, Carroll J, Slagstad D, Ellingsen I, Wassmann P. Intra-regional comparison of productivity, carbon flux and ecosystem composition within the northern Barents Sea. Progress in Oceanography. 2011;90(1):33–46.

[92] Nöthig E-M, Bracher A, Engel A, Metfies K, Niehoff B, Peeken I, et al Summertime plankton ecology in Fram Strait-a compilation of long-and short-term observations. Polar Research. 2015;34.

[93] Mackas D, Sefton H. Plankton species assemblages off southern Vancouver Island: Geographic pattern and temporal variability. Journal of Marine Research. 1982;40(4):1173–200.

[94] Smith SL. Copepods in Fram Strait in summer: distribution, feeding and metabolism. Journal of Marine Research. 1988;46:145–81.

[95] Spies A. Phytoplankton in the marginal ice zone of the Greenland Sea during summer, 1984. Polar biology. 1987;7(4):195–205.

[96] Gradinger R, Baumann M. Distribution of phytoplankton communities in relation to the large-scale hydrographical regime in the Fram Strait. Marine Biology. 1991;111(2):311–21.

[97] Kubiszyn A, Piwosz K, Wiktor J. The effect of inter-annual Atlantic water inflow variability on the planktonic protist community structure in the West Spitsbergen waters during the summer. Journal of Plankton Research. 2014.

[98] Saiz E, Calbet A, Isari S, Anto M, Velasco EM, Almeda R, et al Zooplankton distribution and feeding in the Arctic Ocean during a *Phaeocystis pouchetii* bloom. Deep Sea Research Part I. 2013;72:17–33.

[99] Piwosz K, Walkusz W, Hapter R, Wieczorek P, Hop H, Wiktor J. Comparison of productivity and phytoplankton in a warm (Kongsfjorden) and a cold (Hornsund) Spitsbergen fjord in mid-summer 2002. Polar Biology. 2009;32(4):549–59.

[100] Seuthe L, Iversen KR, Narcy F. Microbial processes in a high-latitude fjord (Kongsfjorden, Svalbard): II. Ciliates and dinoflagellates. Polar Biology. 2011;34(5):751–66.

[101] Comeau AM, Li WK, Tremblay J-É, Carmack EC, Lovejoy C. Arctic Ocean microbial community structure before and after the 2007 record sea ice minimum. PLoS ONE 6(11): e27492 10.1371/journal.pone.0027492 22096583 PMC3212577

[102] Mayzaud P, Boutoute M, Noyon M, Narcy F, Gasparini S. Lipid and fatty acids in naturally occurring particulate matter during spring and summer in a high arctic fjord (Kongsfjorden, Svalbard). Marine Biology. 2013;160(2):383–98.

[103] Beaugrand G, Reid PC, Ibañez F, Planque B. Biodiversity of North Atlantic and North Sea calanoid copepods. Marine Ecology Progress Series. 2000;204:299–303.

[104] Folt CL, Burns CW. Biological drivers of zooplankton patchiness. Trends in Ecology & Evolution. 1999;14:300–5. 10407426 10.1016/s0169-5347(99)01616-x

[105] Wiebe PH, Benfield MC. From the Hensen net toward four-dimensional biological oceanography. Progress in Oceanography. 2003;56:7–136.

[106] Cherkasheva A, Bracher A, Melsheimer C, Köberle C, Gerdes R, Nöthig E-M, et al Influence of the physical environment on polar phytoplankton blooms: a case study in the Fram Strait. Journal of Marine Systems. 2014;132:196–207.

[107] Nilsen F, Gjevik B, Schauer U. Cooling of the West Spitsbergen Current: Isopycnal diffusion by topographic vorticity waves. Journal of Geophysical Research: Oceans. 2006;111(C8).

[108] Teigen SH, Nilsen F, Gjevik B. Barotropic instability in the West Spitsbergen Current. Journal of Geophysical Research: Oceans. 2010;115(C7).

[109] Speirs DC, Gurney WS, Heath MR, Horbelt W, Wood SN, De Cuevas BA. Ocean-scale modelling of the distribution, abundance, and seasonal dynamics of the copepod Calanus finmarchicus. Marine Ecology Progress Series. 2006;313(173–192).

[110] Franks PJ. Sink or swim: Accumulation of biomass at fronts. Marine Ecology Progress Series. 1992;82:1–12.

[111] Labat J-P, Gasparini S, Mousseau L, Prieur L, Boutoute M, Mayzaud P. Mesoscale distribution of zooplankton biomass in the northeast Atlantic Ocean determined with an optical plankton counter: relationships with environmental structures. Deep Sea Research I. 2009;56(10):1742–56.

[112] Kwasniewski S, Gluchowska M, Jakubas D, Wojczulanis-Jakubas K, Walkusz W, Karnovsky N, et al The impact of different hydrographic conditions and zooplankton communities on provisioning Little Auks along the West coast of Spitsbergen. Progress in Oceanography. 2010;87:72–82.

[113] Godø OR, Samuelsen A, Macaulay GJ, Patel R, Hjøllo SS, Horne J, et al Mesoscale eddies are oases for higher trophic marine life. PLoS ONE 7(1): e30161 10.1371/journal.pone.0030161 22272294 PMC3260222

[114] Halvorsen E, Tande KS, Høisæter T. Physical and biological factors influencing the seasonal variation in disatributrion of zooplanton across the shelfat Nordvestbaskem, northern Norway, 1994. Sarsia. 1999;84:279–92.

[115] Loeng H, Drinkwater K. An overview of the ecosystems of the Barents and Norwegian Seas and their response to climate variability. Deep Sea Research II. 2007;54:2478–500.

[116] Oliver K, Eldevik T, Stevens D, Watson A. A Greenland Sea Perspective on the Dynamics of Postconvective Eddies. Journal of Physical Oceanography. 2008;38:2755–71.

[117] Aarbakke ONS, Fevolden S-E, Weydmann A. Relative summer abundances and distribution of *Pseudocalanus* spp.(Copepoda: Calanoida) adults in relation to environmental variables in the Nordic Seas and Svalbard fjords. Polar Biology. 2017;40:51–59.

