# Supplementary material for: Variations in the structural and functional diversity of zooplankton over vertical and horizontal environmental gradients en route to the Arctic Ocean through the Fram Strait
Source: PLoS One. 2017 Feb 8;12(2):e0171715. doi: 10.1371/journal.pone.0171715 (PMC5298267; doi:10.1371/journal.pone.0171715)
Supplement: S1 Table — LAT – latitudinal sections; LON – longitudinal regions; WL – water layer extent. (DOCX) [file pone.0171715.s001.docx]

| LAT | LON | Station | Latitude [⁰N] | Longitude [⁰E] | Date | WL [m] |
| --- | --- | --- | --- | --- | --- | --- |
| 79°N | SLOPE | EB-3 | 78.8401 | 8.4028 | 2012-07-21 | 0-25-50-200-500-670 |
|  | SLOPE | EB-4 | 78.8339 | 8.0685 | 2012-07-21 | 0-25-50-200-600-920 |
|  | SLOPE | EB-5 | 78.8328 | 7.5517 | 2012-07-20 | 0-25-50-200-600-1000 |
|  | SLOPE | EB-6 | 78.8324 | 7.0723 | 2012-07-20 | 0-25-50-200-600-1000 |
|  | OFFSHORE | EB-9 | 78.8215 | 5.5255 | 2012-07-20 | 0-25-50-200-600-1000 |
|  | OFFSHORE | EB-10 | 78.8339 | 5.0049 | 2012-07-20 | 0-25-50-200-600-1000 |
|  | EXTERIOR | EB-15 | 78.8335 | 0.0041 | 2012-07-19 | 0-25-50-200-600-1000 |
|  | EXTERIOR | EB-16 | 78.8321 | -0.8469 | 2012-07-19 | 0-25-50-200-600-1000 |
| 76°30’N | SLOPE | N2 | 76.4924 | 13.0664 | 2012-07-11 | 0-25-50-200-600-1000 |
|  | SLOPE | N1P | 76.4984 | 12.5196 | 2012-07-11 | 0-25-50-200-600-1000 |
|  | SLOPE | N1 | 76.4984 | 11.9982 | 2012-07-11 | 0-25-50-200-600-1000 |
|  | OFFSHORE | N-2 | 76.5032 | 8.9988 | 2012-07-12 | 0-25-50-200-600-1000 |
|  | OFFSHORE | N-3 | 76.5033 | 8.4881 | 2012-07-12 | 0-25-50-200-600-1000 |
|  | OFFSHORE | N-4 | 76.5046 | 8.0010 | 2012-07-12 | 0-25-50-200-600-1000 |
|  | EXTERIOR | N-9 | 76.5128 | 5.5398 | 2012-07-13 | 0-25-50-200-600-1000 |
|  | EXTERIOR | N-10 | 76.5135 | 5.0592 | 2012-07-13 | 0-25-50-200-600-1000 |
|  | EXTERIOR | N-11 | 76.4967 | 3.6627 | 2012-07-13 | 0-25-50-200-600-1000 |
