# Supplementary material for: Variations in the structural and functional diversity of zooplankton over vertical and horizontal environmental gradients en route to the Arctic Ocean through the Fram Strait
Source: PLoS One. 2017 Feb 8;12(2):e0171715. doi: 10.1371/journal.pone.0171715 (PMC5298267; doi:10.1371/journal.pone.0171715)
Supplement: S2 Table — LAT – latitudinal section; LON – longitudinal region; WL – water layer; df – degrees of freedom; MS – means of squares; √ECV – square root of estimated components of variance; %ECV – percentage of ECV to total variation. Bold values denote significance at p<0.05. (DOCX) [file pone.0171715.s002.docx]

| Factor | d*f* | MS | Pseudo-F | *p* | √ECV | ECV% | MS | Pseudo-F | *p* | √ECV | ECV% |
| --- | --- | --- | --- | --- | --- | --- | --- | --- | --- | --- | --- |
|  |  | **Temperature** | | | | | **Salinity** | | | | |
| LAT | 1 | 0.3 | 2.15 | 0.149 | 0.06 | 1.1 | 2.4 | 2.47 | **0.046** | 0.29 | 7.9 |
| LON | 2 | 30.8 | 207.27 | **<0.001** | 1.08 | 18.1 | 0.8 | 0.82 | 0.456 | 0.00 | 0.0 |
| WL | 4 | 67.6 | 454.48 | **<0.001** | 2.07 | 34.9 | 63.7 | 65.18 | **<0.001** | 1.99 | 53.3 |
| LATxLON | 2 | 0.6 | 3.76 | **0.027** | 0.17 | 2.9 | 1.3 | 1.35 | 0.270 | 0.18 | 4.8 |
| LATxWL | 4 | 1.8 | 12.29 | **<0.001** | 0.46 | 7.8 | 0.1 | 0.13 | 0.973 | 0.00 | 0.0 |
| LONxWL | 8 | 2.6 | 17.43 | **<0.001** | 0.68 | 11.4 | 0.5 | 0.51 | 0.847 | 0.00 | 0.0 |
| LATxLONxWL | 8 | 2.9 | 19.72 | **<0.001** | 1.02 | 17.2 | 1.2 | 1.22 | 0.311 | 0.28 | 7.6 |
| Residual | 54 | 0.1 |  |  | 0.39 | 6.5 | 1.0 |  |  | 0.99 | 26.4 |
| Total | 83 |  |  |  |  | 100.0 |  |  |  |  | 100.0 |
|  |  | **Chlorophyll *a*** | | | | | **Total protist biomass** | | | | |
| LAT | 1 | <0.1 | 0.55 | 0.490 | 0.00 | 0.0 | 1315600.0 | 0.92 | 0.362 | 0.00 | 0.0 |
| LON | 2 | <0.1 | 1.12 | 0.367 | 0.04 | 13.2 | 1622500.0 | 1.14 | 0.349 | 192.72 | 13.9 |
| LATxLON | 2 | <0.1 | 0.08 | 0.916 | 0.00 | 0.0 | 1242500.0 | 0.87 | 0.434 | 0.00 | 0.0 |
| Residual | 11 | <0.1 |  |  | 0.28 | 86.8 | 1421900.0 |  |  | 1192.5 | 86.1 |
| Total | 16 |  |  |  |  | 100.0 |  |  |  |  | 100.0 |
|  |  |  | **Protist community structure** | | | |  |  |  |  |  |
| LAT | 1 | 7381.7 | 18.14 | **0.002** | 29.53 | 56.6 |  |  |  |  |  |
| LON | 2 | 439.3 | 1.08 | 0.406 | 2.45 | 4.7 |  |  |  |  |  |
| LATxLON | 2 | 174.5 | 0.43 | 0.870 | 0.00 | 0.0 |  |  |  |  |  |
| Residual | 11 | 407.0 |  |  | 20.18 | 38.7 |  |  |  |  |  |
| Total | 16 |  |  |  |  | 100.0 |  |  |  |  |  |
