# Supplementary material for: Variations in the structural and functional diversity of zooplankton over vertical and horizontal environmental gradients en route to the Arctic Ocean through the Fram Strait
Source: PLoS One. 2017 Feb 8;12(2):e0171715. doi: 10.1371/journal.pone.0171715 (PMC5298267; doi:10.1371/journal.pone.0171715)
Supplement: S3 Table — LAT – latitudinal section; LON – longitudinal region; WL – water layer; df – degrees of freedom; MS – means of squares; √ECV – square root of estimated components of variance; %ECV – percentage of ECV to total variation. Bold values indicate p<0.05. (DOCX) [file pone.0171715.s003.docx]

Table S3

| Factor | d*f* | MS | Pseudo-F | *P* | √ECV | ECV% | MS | Pseudo-F | *p* | √ECV | ECV% | MS | Pseudo-F | *p* | √ECV | ECV% |
| --- | --- | --- | --- | --- | --- | --- | --- | --- | --- | --- | --- | --- | --- | --- | --- | --- |
|  |  | **Standing stock – TA** | | | | | **Standing stock – TB** | | | | | **Zooplankton community structure** | | | | |
| LAT | 1 | 2.4 | 2.47 | **0.046** | 0.29 | 7.8 | 2.8 | 2.5 | 0.108 | 0.21 | 5.4 | 4705.3 | 17.39 | **<0.001** | 10.61 | 12.5 |
| LON | 2 | 0.8 | 0.81 | 0.456 | 0.00 | 0 | 1.1 | 0.9 | 0.387 | 0.00 | 0.0 | 1962.5 | 7.25 | **<0.001** | 7.96 | 9.4 |
| WL | 4 | 63.7 | 65.18 | **<0.001** | 1.99 | 53.3 | 67.4 | 60.9 | **<0.001** | 2.05 | 54.2 | 11191.0 | 41.36 | **<0.001** | 26.31 | 31.1 |
| LATxLON | 2 | 1.3 | 1.35 | 0.270 | 0.18 | 4.8 | 1.4 | 1.3 | 0.280 | 0.16 | 4.2 | 1005.6 | 3.72 | **<0.001** | 7.43 | 8.7 |
| LATxWL | 4 | 0.1 | 0.13 | 0.973 | 0.00 | 0 | 0.8 | 0.7 | 0.620 | 0.00 | 0.0 | 938.7 | 3.47 | **<0.001** | 9.21 | 10.8 |
| LONxWL | 8 | 0.5 | 0.51 | 0.847 | 0.00 | 0 | 1.0 | 0.9 | 0.540 | 0.00 | 0.0 | 507.2 | 1.87 | **<0.001** | 6.67 | 7.8 |
| LAxLOxWL | 8 | 1.2 | 1.22 | 0.311 | 0.28 | 7.6 | 1.4 | 1.2 | 0.281 | 0.31 | 8.3 | 239.7 | 0.88 | 0.719 | 0.00 | 0.0 |
| Residual | 54 | 0.9 |  |  | 0.99 | 26.4 | 1.1 |  |  | 1.06 | 27.8 | 270.6 |  |  | 16.45 | 19.4 |
| Total | 83 |  |  |  |  | 100.0 |  |  |  |  | 100.0 |  |  |  |  | 100.0 |
|  |  | ***C. finmarchicus* - total abundance** | | | | | ***C. finmarchicus* - stage composition** | | | | | ***C. finmarchicus* - stage index** | | | | |
| LAT | 1 | 214350.0 | 1.78 | **0.027** | 48.83 | 5.6 | 4480.3 | 9.28 | **0.001** | 10.07 | 11.74 | 9.5 | 18.98 | **0.002** | 0.48 | 15.2 |
| LON | 2 | 337990.0 | 2.81 | **0.038** | 90.37 | 10.4 | 2420.3 | 5.01 | **0.001** | 8.52 | 9.94 | 4 | 8.07 | **0.002** | 0.36 | 11.6 |
| WL | 4 | 1070100.0 | 8.89 | **0.001** | 245.36 | 28.3 | 10123.0 | 20.95 | **0.001** | 24.72 | 28.82 | 9.4 | 18.99 | **0.001** | 0.75 | 24 |
| LATxLON | 2 | 125620.0 | 1.04 | 0.394 | 19.86 | 2.3 | 598.6 | 1.24 | 0.322 | 2.94 | 3.43 | 0.1 | 0.11 | 0.919 | 0.00 | 0.0 |
| LATxWL | 4 | 55762.0 | 0.46 | 0.759 | 0 | 0.0 | 1511.9 | 3.13 | **0.003** | 11.42 | 13.31 | 3.5 | 7.05 | **0.001** | 0.62 | 19.7 |
| LONxWL | 8 | 190390.0 | 1.58 | 0.16 | 114.57 | 13.2 | 682.4 | 1.41 | 0.107 | 6.11 | 7.12 | 0.8 | 1.53 | 0.158 | 0.22 | 7.1 |
| LATxLONxWL | 8 | 107890.0 | 0.9 | 0.526 | 0 | 0.0 | 469.6 | 0.97 | 0.510 | 0 | 0.0 | 0.4 | 0.85 | 0.566 | 0.00 | 0.0 |
| Residual | 54 | 120370.0 |  |  | 346.94 | 40 | 483.2 |  |  | 21.98 | 25.63 | 0.5 |  |  | 0.71 | 22.5 |
| Total | 83 |  |  |  |  | 100.0 |  |  |  |  | 100.0 |  |  |  |  | 100.0 |
|  |  | **Species richness - H_o_** | | | | | **Species evenness - H_inf_** | | | | | **Trophic diversity - TD^-1^** | | | | |
| LAT | 1 | <0.1 | 0.01 | 0.971 | 0.00 | 0.0 | <0.1 | 21.21 | **<0.001** | 0.05 | 18.5 | <0.1 | 0.27 | 0.602 | 0.00 | 0.0 |
| LON | 2 | <0.1 | 0.54 | 0.585 | 0.00 | 0.0 | <0.1 | 1.29 | 0.281 | 0.01 | 2.7 | <0.1 | 2.38 | 0.107 | 0.01 | 6.1 |
| WL | 4 | 0.5 | 116.19 | **<0.001** | 0.18 | 51.3 | 0.1 | 29.24 | **<0.001** | 0.09 | 34.5 | <0.1 | 16.57 | **<0.001** | 0.06 | 26.8 |
| LATxLON | 2 | 0.1 | 3.10 | 0.047 | 0.03 | 7.5 | <0.1 | 0.12 | 0.884 | 0.00 | 0.0 | <0.1 | 3.71 | 0.031 | 0.03 | 12.1 |
| LATxWL | 4 | <0.1 | 1.28 | 0.283 | 0.01 | 3.6 | <0.1 | 4.81 | **<0.001** | 0.05 | 17.9 | <0.1 | 2.50 | 0.052 | 0.02 | 11.7 |
| LONxWL | 8 | 0.1 | 2.26 | 0.042 | 0.03 | 9.2 | <0.1 | 0.70 | 0.673 | 0.00 | 0.0 | <0.1 | 2.92 | **<0.001** | 0.03 | 16.2 |
| LATxLONxWL | 8 | <0.1 | 1.64 | 0.132 | 0.03 | 9.3 | <0.1 | 1.00 | 0.451 | 0.01 | 0.6 | <0.1 | 0.82 | 0.585 | 0.00 | 0.0 |
| Residual | 54 | <0.1 |  |  | 0.07 | 18.9 | <0.1 |  |  | 0.07 | 25.8 | <0.1 |  |  | 0.06 | 26.9 |
| Total | 83 |  |  |  |  | 100.0 |  |  |  |  | 100.0 |  |  |  |  | 100.0 |
