# Supplementary material for: Variations in the structural and functional diversity of zooplankton over vertical and horizontal environmental gradients en route to the Arctic Ocean through the Fram Strait
Source: PLoS One. 2017 Feb 8;12(2):e0171715. doi: 10.1371/journal.pone.0171715 (PMC5298267; doi:10.1371/journal.pone.0171715)
Supplement: S4 Table — Var%—percentage of explained variance; Cum%—cumulative percentage explained by the added variable. Bold values denote significance at p<0.05. (DOCX) [file pone.0171715.s004.docx]

| MARGINAL TESTS | | | | SEQUENTIAL TESTS | | | | | |
| --- | --- | --- | --- | --- | --- | --- | --- | --- | --- |
| Variable | ps-F | *p* | var% | Variable | R^2^ | ps-F | *p* | Var% | Cum% |
| **Zooplankton abundance, all layers** | | | |  |  |  |  |  |  |
| Temperature | 47.71 | **0.001** | 0.37 | Temperature | 0.37 | 47.71 | **0.001** | 0.37 | 0.37 |
| Salinity | 1.27 | 0.232 | <0.01 | +Salinity | 0.44 | 10.49 | **0.001** | 0.07 | 0.44 |
| **Zooplankton abundance, avg. over 0-1000 m** | | | |  |  |  |  |  |  |
| Temperature | 6.50 | **0.001** | 0.31 | Temperature | 0.30 | 6.50 | **0.002** | 0.31 | 0.31 |
| Salinity | 1.79 | 0.067 | 0.11 | +Salinity | 0.41 | 2.62 | **0.013** | 0.11 | 0.41 |
| **Herbivorous zooplankton abundance, avg. over 0-50 m** | | | |  |  |  |  |  |  |
| Temperature | 2.63 | **0.042** | 0.15 | Bacillariophyceae | 0.18 | 3.34 | **0.012** | 0.18 | 0.18 |
| Salinity | 1.04 | 0.396 | 0.06 | +Temperature | 0.29 | 2.13 | **0.021** | 0.11 | 0.29 |
| Chlorophyll *a* | 0.39 | 0.814 | 0.26 | +Salinity | 0.49 | 4.96 | **0.009** | 0.19 | 0.48 |
| Bacillariophyceae | 3.34 | **0.008** | 0.18 | +Haptophyta | 0.52 | 1.00 | 0.389 | 0.04 | 0.52 |
| Dinoflagellata | 1.68 | 0.157 | 0.11 | +Flagellates indet. | 0.54 | 0.49 | 0.729 | 0.02 | 0.54 |
| Flagellates indet. | 0.72 | 0.568 | 0.04 | +Dinoflagellata | 0.56 | 0.34 | 0.830 | 0.02 | 0.56 |
| Haptophyta | 1.47 | 0.224 | 0.09 | +Chlorophyll *a* | 0.59 | 0.64 | 0.582 | 0.29 | 0.59 |
